# Planktonic Archaeal Ether Lipid Origins in Surface Waters of the North Pacific Subtropical Gyre

**DOI:** 10.3389/fmicb.2021.610675

**Published:** 2021-09-13

**Authors:** Fuyan Li, Andy Leu, Kirsten Poff, Laura T. Carlson, Anitra E. Ingalls, Edward F. DeLong

**Affiliations:** ^1^Daniel K. Inouye Center for Microbial Oceanography: Research and Education, University of Hawai’i at Mânoa, Honolulu, HI, United States; ^2^School of Oceanography, University of Washington, Seattle, WA, United States

**Keywords:** planktonic Thermoplasmatota, archaeal ether lipids, archaeal GDGT ring synthases, euphotic zone, NPSG

## Abstract

Thaumarchaeota and Thermoplasmatota are the most abundant planktonic archaea in the sea. Thaumarchaeota contain tetraether lipids as their major membrane lipids, but the lipid composition of uncultured planktonic Thermoplasmatota representatives remains unknown. To address this knowledge gap, we quantified archaeal cells and ether lipids in open ocean depth profiles (0–200 m) of the North Pacific Subtropical Gyre. Planktonic archaeal community structure and ether lipid composition in the water column partitioned into two separate clusters: one above the deep chlorophyll maximum, the other within and below it. In surface waters, Thermoplasmatota densities ranged from 2.11 × 10^6^ to 6.02 × 10^6^ cells/L, while Thaumarchaeota were undetectable. As previously reported for Thaumarchaeota, potential homologs of archaeal tetraether ring synthases were present in planktonic Thermoplasmatota metagenomes. Despite the absence of Thaumarchaeota in surface waters, measurable amounts of intact polar ether lipids were found there. Based on cell abundance estimates, these surface water archaeal ether lipids contributed only 1.21 × 10^–9^ ng lipid/Thermoplasmatota cell, about three orders of magnitude less than that reported for Thaumarchaeota cells. While these data indicate that even if some tetraether and diether lipids may be derived from Thermoplasmatota, they would only comprise a small fraction of Thermoplasmatota total biomass. Therefore, while both MGI Thaumarchaeota and MGII/III Thermoplasmatota are potential biological sources of archaeal GDGTs, the Thaumarchaeota appear to be the major contributors of archaeal tetraether lipids in planktonic marine habitats. These results extend and confirm previous reports of planktonic archaeal lipid sources, and further emphasize the need for Thermoplasmatota cultivation, to better characterize the membrane lipid constituents of marine planktonic Thermoplasmatota, and more precisely define the sources and patterns of archaeal tetraether lipid distributions in marine plankton.

## Introduction

Marine planktonic Archaea are abundant in diverse marine planktonic environments (DeLong, 1992, 1998; Fuhrman et al., 1992; DeLong et al., 1994), and play important roles in the biogeochemical cycles of carbon and nitrogen (Ingalls et al., 2006; Falkowski et al., 2008; Martin-Cuadrado et al., 2008; Iverson et al., 2012). Three major groups of planktonic archaea are distributed in the marine water column. The most abundant group was originally called Marine Group I (MGI) planktonic Crenarchaeota (DeLong, 1992). This general phylogenetic group was later referred to as Thaumarchaeota as genomic analyses and protein phylogenies became available (Preston et al., 1996; Hallam et al., 2006a, b; Brochier-Armanet et al., 2008). Thaumarchaeota are more abundant below the base of the epipelagic zone and in the meso- and bathypelagic (Karner et al., 2001; Mincer et al., 2007; Brown et al., 2009; Church et al., 2010). The two other most abundant planktonic archaeal groups belong to the Thermoplasmatota (formerly Thermoplasmatales in Euryarchaeota, Rinke et al., 2019), and include MGII Archaea (DeLong, 1992), which are more abundant in the surface and in the deep chlorophyll maximum (DCM) (Massana et al., 2000; DeLong et al., 2006; Mincer et al., 2007; Hugoni et al., 2013); and MGIII Archaea (Fuhrman and Davis, 1997), which can be detected throughout the water column but are found more frequently in deeper waters (Massana et al., 1997; DeLong et al., 2006; Haro-Moreno et al., 2017). To date, only the Thaumarchaeota have been cultivated, and all cultivars are chemolithotrophic ammonia oxidizers (Könneke et al., 2005; Kim et al., 2016; Qin et al., 2017). Some deeply branching lineages affiliated with Thaumarchaeota pSL12-like clade however (Mincer et al., 2007; Church et al., 2010), appear to be capable of heterotrophic growth (Aylward and Santoro, 2020; Reji and Francis, 2020). Symbiotic or cultured MGI Thaumarchaeota synthesize glycerol dialkyl glycerol tetraethers (GDGTs) with zero through four cyclopentane rings (DeLong et al., 1998; Schouten et al., 2008) and crenarchaeol, a distinct GDGT containing four cyclopentane and one cyclohexane ring (Schouten et al., 2008). Among isolated Archaea, crenarchaeol is unique to Thaumarchaeota and is also ubiquitous within this group. As such, crenarchaeol is used extensively as a biomarker for ammonia-oxidizing Archaea (AOA) (Schouten et al., 2008, 2013; Qin et al., 2015; Kim et al., 2016). The other GDGTs with cyclopentane rings are also synthesized by other archaea including members of the Crenarchaeota and Euryarchaeota (Pearson and Ingalls, 2013).

Glycerol dialkyl glycerol tetraethers produced by planktonic Thaumarchaeota within and below the DCM (primarily between 80 and 250 m) are incorporated into sinking particles, and accumulate in sediments (Hurley et al., 2018). Intact polar lipids (IP-GDGTs), core lipids (C-GDGTs) with one to two phosphatidic, glycosidic, or glycophosphatidic head groups attached, are assumed to rapidly degrade with cleavage of polar head groups after cell death (White et al., 1979; Harvey et al., 1986), and are used as quantitative indicators of living AOA in the marine water column (Schubotz et al., 2009; Pitcher et al., 2011; Ingalls et al., 2012; Basse et al., 2014). C-GDGTs are more recalcitrant to degradation and can be preserved in sediments over millions of years, which enables the application of these lipids in paleoenvironmental studies (Kuypers et al., 2001; Pearson et al., 2001). The sediment core-top TEX_86_ proxy, based on the ratio between C-GDGTs with one to three cyclopentane moieties and the crenarchaeol isomer, is observed to correlate with sea surface temperature (SST) (Schouten et al., 2002; Kim et al., 2010). This proxy is used to reconstruct the paleotemperature of the sea surface (Zachos et al., 2006; Tierney et al., 2010; Schefuß et al., 2011). MGI Thaumarchaeota and their lipids occur preferentially in the subsurface and deeper, however, where temperatures are much lower than SST, especially from the tropical to subtropical regions (∼30°N–30°S) (Ho and Laepple, 2016; Hurley et al., 2018; Zhang and Liu, 2018).

The mismatch between the habitat and depth profiles of known GDGT producing Thaumarchaeota and the use of GDGTs as a proxy of SST, has led to speculation that other planktonic archaeal groups, in particular the Thermoplasmatota, may also produce GDGTs in surface waters and contribute to sedimentary GDGTs (Lincoln et al., 2014; Ma et al., 2020). It was proposed that the prevalence of MGII Archaea at the ocean’s surface might help explain why surficial sediment TEX_86_ values reflect SST temperatures, even though most modern-day Thaumarchaeota do not reside in the sea surface and their lipids are only present in trace quantities. The TEX_86_ signatures of Thaumarchaeota could also be influenced by oxygen availability (Qin et al., 2015) and ammonia oxidation rate (Hurley et al., 2016). Environmental metagenome assembly data show that genomes of MGII/III Archaea harbor genes coding for catalysis of ether bond formation (i.e., geranylgeranylglyceryl phosphate synthase, GGGP; digeranylgeranylglyceryl phosphate synthase, DGGGP), and that these groups of archaea may have the genetic potential to synthesize GDGTs (Iverson et al., 2012; Deschamps et al., 2014; Villanueva et al., 2017). Station ALOHA offers an opportunity to use a well-stratified water column to directly compare lipids and genes.

**FIGURE 1:**
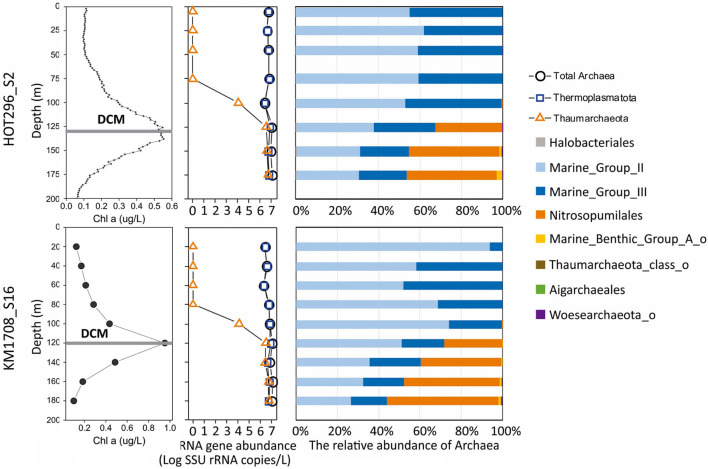
Distribution and abundance of different archaeal groups and Chl a in the water column as a function of depth. The line plots **(left)** show distribution patterns of Chl a and absolute archaeal rRNA gene abundances as a function of depth. The bar plots **(right)** show the relative abundance of the different archaeal lineages as a function of depth.

Current best estimates for the origin of the TEX_86_ proxy indicate that archaeal lipids produced at or near the base of the euphotic zone are the major contributors to sedimentary GDGTs (Hurley et al., 2018). This also often corresponds with the base of the nitrite maximum. The euphotic layer of the North Pacific Subtropical Gyre (NPSG) is on average at ∼173 m (±7 m) (Letelier et al., 1996; Karl and Church, 2017). The upper primary nitrite maximum is located within the DCM or just below it, i.e., near the base of the euphotic layer (Dore and Karl, 1996). To further investigate the contribution of MGI Thaumarchaeota and MGII/III Thermoplasmatota to the production of ether lipids in the euphotic zone, we examined planktonic archaeal community profiles determined by amplicon sequence analyses, and the distribution of ether lipids (i.e., GDGTs and archeol) in high resolution depth profiles (between 0 and 200 m) in the NPSG (Figure 1 and Supplementary Table 1). Amplicon sequencing, and droplet digital Polymerase Chain Reaction (ddPCR) analyses of small subunit ribosomal RNA (SSU rRNA) genes, were used to determine archaeal community profiles in the upper 200 m. Using the same filter samples, we measured in parallel ether lipids including C-, monohexose- (MH-GDGTs), dihexose- (DH-GDGTs), phosphohexose- (PH-GDGTs), and hexose-phosphohexose-GDGTs (HPH-GDGTs), and C-archeol (see structures in Supplementary Figure 1). We explore the relationship between GDGT occurrence and planktonic archaeal communities found at the same depths.

## Materials and Methods

### Sampling and Water Parameters

Seawater samples from the euphotic zone (between surface and 200 m) were collected at the Hawaii Ocean Time-Series Station ALOHA [Station 2 (S2)] during HOT cruise 296 (October 5–8, 2017), and at the center of a cyclonic eddy [Station 16 (S16)] during the MESO-SCOPE cruise KM1709 (June 26 to July 15, 2017) (Supplementary Figure 2 and Supplementary Table 1). The filtration was subsequently conducted through 0.22 μm Durapore PVDF membrane filters with 25 mm diameter for DNA sample collection and 47 mm diameter for lipid sample collection (EMD Millipore, Billerica, MA, United States) (see detailed information in Supplementary Table 1). Samples of 2 L were collected for DNA analysis whereas samples of 20–30 L from the remaining water were collected for lipid analysis. After filtering was completed, DNA filter samples were immediately placed in RNAlater stabilization solution (Ambion, Carlsbad, CA, United States). All filters were frozen at −80°C on board, then transported to lab using dry ice and preserved at −80°C prior to extraction of DNA and lipids.

Environmental parameters such as temperature, salinity, density and Chl a were retrieved from CTD data available on the HOT-DOGS website http://hahana.soest.hawaii.edu/hot/hot-dogs/ for station HOT296_S2 and on the SCOPE website http://scope.soest.hawaii.edu/data/mesoscope/mesoscope.html for KM1709_S16.

### DNA Extraction, Sequencing and Quantification

DNA was extracted using the PowerBiofilm DNA Isolation kit (MO BIO, Carlsbad, CA, United States) according to the manufactures protocol after RNAlater was removed. The V4 region of the SSU rRNA gene was amplified with universal primers 515F (5′-GTGYCAGCMGCCGCGGTAA-3′, Parada et al., 2016) and 806RB (5′-GGACTACNVGGGTWTCTAAT-3′, Apprill et al., 2015), modified from Caporaso et al. (2011). PCR amplification procedures were modified from the SSU rRNA illumina amplicon protocol of Earth Microbiome Project (2018). Cycling conditions are as follows: 94°C for 3 min; 28 cycles of 94°C for 45 s, 50°C for 60 s, and 72°C for 90 s; followed by 72°C for 10 min. Amplicons were purified with AMPure XP beads (Beckman Coulter, Brea, CA, United States) and their size was verified using agarose gel electrophoresis. Following this step, all purified PCR products were pooled together and sequenced on the illumina MiSeq platform using the MiSeq Reagent Kits v2 300-cycles chemistry (Illumina, San Diego, CA, United States). All SSU rRNA amplicon sequencing data have deposited in the NCBI Sequence Read Archive under project number PRJNA614540.

The ddPCR assays were performed with the same barcoded primers. Briefly, the reaction mixtures contained 1 × EvaGreen ddPCR Supermix (Bio-Rad, Hercules, CA, United States), 100 nM of each primer and 0.03 ng of template DNA in 20 μL volume. Control reactions replaced the DNA template with nuclease-free water (Ambion, Austin, TX, United States). Each reaction was mixed with 70 μL of droplet generation oil (Bio-Rad) and partitioned into 14657–19526 droplets in a QX200 droplet generator (Bio-Rad). After processing, droplets were transferred into a 96-well ddPCR plate (Bio-Rad) and heated-sealed with pierceable foil (Bio-Rad). The PCRs were initiated at 95°C for 5 min DNA polymerase activation, followed by 40 cycles of a two-step thermal profile of 30 s at 95°C for denaturation and 2 min at 54.6°C for annealing and extension, and one cycle of 4°C for 5 min and 90°C for 5 min for droplet stabilization. After amplification, the fluorescence intensity of individual droplets was detected with QX200 droplet reader (Bio-Rad). Data were analyzed with QuantaSoft droplet reader software (Bio-Rad). Positive and negative signals were detected automatically or manually and the target DNA concentrations were calculated. Nothing was obtained in control reaction.

### Processing of Sequence Data

Illumina pair-end sequences were processed with Trimmomatic v0.36 (Bolger et al., 2014), QIIME 1.9.1 (Caporaso et al., 2010), and DADA2 v1.6 (Callahan et al., 2016). Trimmomatic was used to remove adapters and trim low quality bases using the associated threshold parameters: the minimum leading quality = 3, the minimum trailing quality = 3, the number of bases within sliding window = 4, the average quality required for sliding window = 15, the minimum length = 100. The trimmed reads were demultiplexed and split into individual sample files with QIIME. After trimming and demultiplexing, amplicon sequences were further processed using the R package DADA2. We removed any sequences with length shorter than 110 or longer than 220, with ambiguous nucleotide assignment, or with greater than 2 expected errors. All identical sequences were combined into unique sequences through the step of dereplication. An error model was generated to evaluate the probability of each unique sequence being a real sequence versus an artifact sequence produced from PCR or sequencing. The error model was subsequently parameterized and applied for denoising forward and reverse reads independently. The denoised forward and reverse reads were merged together with overlap by at least 12 bases. Amplicon sequence variants (AASVs) were constructed based on exact sequence variants inferred from merged sequences, and then chimeras were removed. The taxonomy was assigned to individual AASVs using the SILVA v132 database. Only sequences restricted to Archaea were included for the following analysis and discussion in this study.

### Phylogenetic Analysis

BLASTP searches of archaeal GDGT ring synthases GrsA (Saci_1585) and GrsB (Saci_0240), and Saci_1785 (as an outgroup) homologs from archaeal isolates in Supplementary Table 1 of Zeng et al. (2019) was performed against the Genome Taxonomy Database using an *e*-value cutoff of ≤1e^–5^ and amino acid identity cutoff of ≥20%. Hits against reference MGII and MGIII genomes were collected. In addition, the ALOHA gene catalog (Mende et al., 2017) was searched for putative GrsA and GrsB homologs from MGII and MGIII with an amino acid identity cutoff of ≥90%.

The Grs homologs from MGII and MGIII metagenome assembled genomes (MAGs) in GTDB and the ALOHA gene catalog, and from the archaeal isolates were filtered by length (≥400 aa) and aligned using mafft v7.407 with the–auto function and the alignment trimmed using trimal v1.4^1^ “-automated1’’ option. A phylogenetic tree was constructed using FastTree V. 2.1.10 with the LG + GAMMA models and rooted by midpoint. Bootstrap values were calculated via non-parametric bootstrapping with 100 replicates. The tree was visualized using the Interactive Tree of Life (iTOL) online tool^2^ (Letunic and Bork, 2016).

The metagenomic sequence read coverage value for the putative Grs homologs of MGII/III from the ALOHA gene catalog 2.0 was estimated using coverM 0.3.1 with the -m trimmed_mean –min-read-percent-identity 0.95 –min-read-aligned-percent 0.75 option. The coverage profile was reported here with the cutoff of 1.

### Lipid Extraction and Analysis

Lipids were ultrasonically extracted from filters using a modified Bligh-Dyer method with a solvent mixture of methanol, dichloromethane and aqueous buffer (2:1:0.8) (Sturt et al., 2004). Phosphate buffer (50 mM) was used for the first two extractions and trichloroacetic acid buffer (50 mM) was used for the remaining two extractions (Sturt et al., 2004). The total lipid extracts (TLEs) were dried under a stream of N_2_, dissolved in methanol with the addition of the internal standard C_46_ and then split into two equal aliquots. One aliquot was subjected to acid hydrolysis with 5% HCl in methanol at 100°C overnight to cleave polar head groups.

Both non-hydrolyzed and hydrolyzed aliquots were analyzed with a Waters I-class Acquity ultra-high performance liquid chromatograph (Waters, Milford, MA, United States) coupled to a Q Exactive HF Orbitrap mass spectrometer (UHPLC-ESI-MS_*Q* Exactive_ Thermo Fisher Scientific, Waltham, MA, United States) equipped with an electrospray ionization source. Samples were run with the mass spectrometer in full scan mode (450–1800 m/z) and again in selected ion monitoring (SIM) mode in order to enhance sensitivity for very low abundance lipids detected in the full scan mode. Analyte separation was achieved on an ACE 3 C18 column (2.1 × 150 mm, 3 um particle size, Advanced Chromatography Technologies Ltd., Aberdeen, Scotland) maintained at 45°C with a flow rate of 0.2 mL/min (Zhu et al., 2013). Solvent A was methanol, formic acid, and ammonia (100/0.04/0.10) and Solvent B was Isopropyl alcohol, formic acid, ammonia (100/0.04/0.10). In brief, GDGTs were eluted with the following gradient: 100% A for 1 min, followed by a gradient to 24% B over 5 min and a gradient to 65% B over 55 min. The column was flushed with 75% B for 5 min and re-equilibrated with 100% A for 8 min.

In order to minimize the noise and collect enough data points per peak based on the Q Exactive resolution, multiple time windows related to each lipid class were applied in the selected ion monitoring (SIM), with each time window having the maximum of 20 compounds targeted using the scan mass point of 0.5 Da. Target lipids (Supplementary Figure 1) were determined with SIM [i.e., ammoniated ([M + NH_4_]^+^), sodiated ([M + Na]^+^), and protonated ([M + H]^+^) molecules]. The further MS/MS fragmentation was carried out only for [M + NH_4_]^+^ and [M + H]^+^ molecules on selected samples using collision energy 20 or 25.

Glycerol dialkyl glycerol tetraethers and archeol were identified by retention time, molecular mass and MS/MS fragmentation patterns. Corresponding core lipids before and after acid hydrolysis were quantified relative to the response of the internal standard C_46_. A standard mix solution was constructed with C_46_, the purchased archeol standard (1,2-di-O-phytanyl-sn-glycerol from Avanti Lipids, Alabaster, AL, United States), and crenarchaeol which was purified from a marine sediment extract in Ann Pearson’s lab in Harvard University. Dilution series of the standard mix solution ranged in concentration from 1 to 5 ng of each standard. Response factors of 1.5 for crenarchaeol and 4.0 for archeol were applied to core lipids, based on the averaged relative response of crenarchaeol and archeol relative to the C_46_ internal standard, respectively.

Only compounds with signal to noise ratio (SN) ≥ 5 (calculated automatically in the Xcalibur with the algorithm ICIS) were reported. Due to the lack of authentic internal standards, absolute quantification of MH-, DH-, PH-, and HPH-GDGTs can’t be calculated. Instead, relative distribution of these GDGTs in individual intact polar lipid forms was reported separately here based on the peak area. Abundances of IP-GDGTs and -archeol were obtained by subtraction of core lipids between non-hydrolyzed and hydrolyzed fractions (Ingalls et al., 2012). When using this hydrolysis method, negative calculated values of intact polar lipids were obtained in the surface, likely due to differences in matrix effects related to variability in sample preparation. We treated the negative value as zero for individual IP-ethers when calculating the sum of IP-ethers, and only reported the concentration of total IP-ethers for each sample when comparing with the archaeal biomass (see Supplementary Table 1).

## Results

Water column profiles in the epipelagic zone in the NPSG were investigated from the HOT Station ALOHA (S2) during HOT cruise 296 and from the center of a cyclonic eddy (S16) during the MESO-SCOPE cruise KM1709 (Supplementary Figure 2). The hydrographic features (temperature, salinity, density, and chlorophyll a) were in general similar between the two samplings (Supplementary Figure 2). Station HOT296_S2 had a shallow salinity maximum at around 140 m, due to the subduction of high salinity waters into the pycnocline (Lukas, 2001). The DCM occurred at 120 m in station HOT296_S2, and at 125 m in station KM1709_S16 (Supplementary Figure 2).

### Vertical Profiles of the Archaeal Community Based on Investigation of the SSU rRNA Gene

A total of 162,748 high-quality archaeal sequences were recovered from the two vertical profiles at stations HOT296_S2 and KM1709_S16, which ranged from 1,695 to 40,915 sequences per sample, with an average of 9,573 per sample (Supplementary Table 2). Rarefaction curves of archaeal AASVs detected relative to sequencing effort, displayed clear signs of saturation, suggesting that archaeal diversity was adequately sampled (Supplementary Figure 3). In all, 231 AASVs were identified, which ranged from 18 to 23 AASVs per sample (a mean value of 21) above DCM of HOT296_S2 and from 9 to 18 (a mean value of 14) above DCM of KM1709_S16 (Supplementary Figure 4A). The richness observed within and below DCM varied between 33 and 78 AASVs per sample (a mean value of 52) in HOT296_S2, and 48–154 (a mean value of 105) AASVs in KM1709_S16 (Supplementary Figure 4A). The Shannon index estimated alpha diversity ranged from 2.34 to 2.82 (a mean value of 2.54) above the DCM in HOT296_S2, and from 1.82 to 2.40 (a mean value of 2.04) in KM1709_S16 (Supplementary Figure 4B). Within and below DCM, the Shannon index varied between 2.33 and 3.14 (a mean value of 2.66) in HOT296_S2 and between 2.82 and 4.08 (a mean value of 3.63) in KM1709_S16 (Supplementary Figure 4B). Diversity estimates of richness and Shannon index both were generally lower above DCM, relative to those within and below DCM.

Archaeal community structure varied with depth. Above the DCM, Thermoplasmatota were by far the predominant, if not exclusive, planktonic archaeal group present by several orders of magnitude. Thaumarchaeota represented less than 0.5% Archaea at 100 m at both stations, Halobacterota [the phylum proposed based on genome phylogenies (Parks et al., 2018)^3^ ] represented less than 0.4% Archaea at 75 and 100 m at the HOT296_S2 station and Nanoarchaeota represented less than 0.1% of Archaea at 25 and 45 m at the HOT296_S2 station (Figure 1 and Supplementary Table 2). Within and below the DCM, planktonic Thermoplasmatota accounted for 53.3–67.2% of the total Archaea in HOT296_S2 and 43.9–71.8% in KM1709_S16. Thaumarchaeota were significantly enriched in and below the DCM, comprising 32.2–46.1% of archaeal rRNAs in HOT296_S2, and 28.2–55.2% in KM1709_S16 (Figure 1 and Supplementary Table 2). Planktonic Thermoplasmatota were dominated by MGII (51.9–93.9% Archaea above DCM, and 26.4–51.0% Archaea within and below DCM) being relatively more abundant, compared to MGIII (6.1–48.1% Archaea above DCM, and 17.6–30.0% Archaea within and below DCM) (Figure 1 and Supplementary Table 2).

**FIGURE 2:**
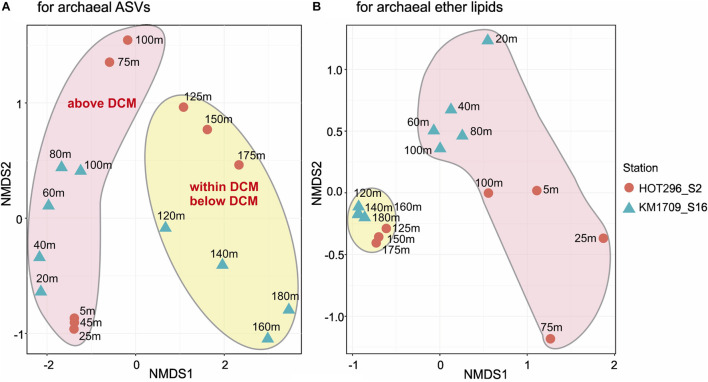
Non-metric multidimensional scaling plots of archaeal rRNA and lipid abundance patterns. Bray-Curtis distance matrices generated from relative abundances of archaeal ASVs **(A)** and ether lipids **(B)**. Both stress values (0.063 for archaeal ASVs and 0.055 for archaeal GDGTs) are less than 0.1, indicating that each of the NMDS ordinations significantly represents the configuration of samples.

Non-metric multidimensional scaling (NMDS) analysis was used to visualize Bray-Curtis dissimilarity profiles of AASV distributions among the different samples. NMDS plots showed the tendency of samples within and below the DCM to be more tightly clustered, and separated from samples above the DCM (Figure 2A).

### Depth Distribution of Archaeal Ether Lipids

**FIGURE 3:**
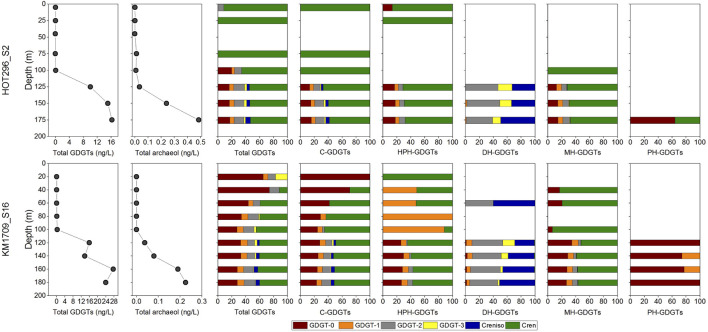
Archaeal lipid distributions and concentrations in the water column. Total GDGTs (the sum of C- and IP-GDGTs) and archeol (the sum of C- and IP-archeol) from acid-hydrolyzed fraction, and relative abundances of individual GDGTs in total lipids and in C-, HPH-, DH-, MH-, and PH-GDGTs along depths.

Hexose- phosphohexose-, PH-, DH-, MH-, and C-GDGTs and C-archeol (Supplementary Figure 1) were identified here and their structures were confirmed by MS/MS analysis (see Supplementary Text and Supplementary Figures 5–10). Although we did not determine a limit of detection or quantification for each GDGT, a peak that was five times the noise (a reasonable value for a limit of quantification using SIM) was estimated to be 0.00060 ng/L (crenarchaeol isomer in the acid-hydrolyzed fraction at 100 m in KM1709_S16) and was the lowest concentration we calculated. The quantification limit was applied to core lipids of GDGTs detected in hydrolyzed and non-hydrolyzed fractions. The peak for GDGT-1 at 20 m in KM1709_S16 had a SN close to 5 and was therefore less than the limit of quantification of 0.0006 ng/L. This compound however, was likely a real peak showing the presence of GDGT-1, and thus was reported here. All sampling depths showed the existence of GDGTs, except the 45 m station of HOT296_S2 where GDGTs were below the quantification limit (0.00060 ng/L) (Figure 3 and Supplementary Figure 5). In this sample, a mass feature with the same exact mass as GDGT-0 was detected after acid hydrolysis of the sample, but it could not be differentiated from noise (signal to noise ratio = 2, Supplementary Figure 5). This lipid sample could be treated as the control collected *in situ*, compared to other lipid samples. Subsequent GDGT analyses discussed here therefore refer to all but this one sample. The identity of core and intact polar lipids within shallow depths (≤40 m) was validated by detection of characteristic fragment ions in MS/MS spectra (see Supplementary Figures 11–14). The intensity of the mass spectral signal was lower in shallow samples compared to deep ones (e.g., those below DCM), but dominant product ions were still observed in near-surface samples (Supplementary Figures 11–14).

Hexose- phosphohexose-, PH-, DH-, MH-, and C-GDGTs and C-archeol were detected in the non-hydrolyzed fractions while total GDGTs and total archeol were detected in acid hydrolyzed fractions (Figure 3). Above the DCM, concentrations of total GDGTs were very low, ranging from 0.015 to 0.077 ng/L in HOT296_S2 and from 0.0058 to 0.19 ng/L in KM1709_S16 (Figure 3, Supplementary Table 1, and Supplementary Figure 15). GDGTs under the quantification limit (0.00060 ng/L) would not have a significant impact on the GDGT distributions at the surface (see the measured concentrations of individual total GDGTs compared to the limit in Supplementary Table 1). Within and below the DCM, total GDGT concentrations increased greatly by 100–1000 fold, and varied between 6.73 and 11.32 ng/L in HOT296_S2, and 9.22–18.59 ng/L in KM1709_S16 (Figure 3, Supplementary Table 1, and Supplementary Figure 15). Along depth profiles, the total GDGTs reached maximum values at 175 m at the HOT296_S2 station, and at 160 m at the KM1709_S16 station (Figure 3, Supplementary Table 1, and Supplementary Figure 15). Profiles of total archeol showed the same pattern in depth profiles at both stations, except that the maximum value at the KM1709_S16 station was at 180 m (Figure 3, Supplementary Table 1, and Supplementary Figure 15). Archeol was either undetectable, or accounted for only a minor fraction of archaeal lipids (mostly less than 5%), at all depths except 75 m of HOT296_S2, where the diethers comprised 78% of all archaeal ether lipids (Figure 3, Supplementary Table 1, and Supplementary Figure 15). For the intact polar counterparts of archeol, only monohexose-archeol was detected in our samples.

In the total GDGT pool and in the C-, HPH-, and MH-GDGTs pools, crenarchaeol was either the only compound, or the predominant compound detected above the DCM in HOT296_S2. Other GDGTs were present in lesser abundance, or below detection limits. For example, GDGT-2 from hydrolyzed fractions and GDGT-0 in the HPH-GDGTs were at lower abundance at 5 m depth (Figure 3). The distributional patterns of GDGTs above DCM in KM1709_S16 were more complex. In the total GDGTs and in C-GDGTs, GDGT-0 was the most abundant molecule at 20 and 40 m, whereas samples at 60, 80, and 100 m were dominated by GDGT-0 and crenarchaeol (Figure 3). In the HPH-GDGT fraction, crenarchaeol was the only lipid detected at 20 m, GDGT-1 was predominant at 80 and 100 m, and samples at 40 and 60 m were composed of almost equal GDGT-1 and crenarchaeol (Figure 3). GDGT-2 and crenarchaeol isomer were observed abundantly at 60 m in the DH-GDGT fraction (Figure 3). The MH-GDGT patterns above the DCM were more similar to those found at the HOT296_S2 station, and were dominated by crenarchaeol (Figure 3).

Within and below the DCM, GDGTs at the HOT296_S2 station closely resembled those of KM1709_S16, and contained a high abundance of GDGT-0 and crenarchaeol in the total GDGTs and in C-, HPH-, and MH-GDGT fractions, with GDGT-2 and the crenarchaeol isomer dominating the DH-GDGTs, and GDGT-0 being the most abundant lipid in PH-GDGTs (Figure 3).

Non-metric multidimensional scaling analyses of the depth distributions of GDGTs and archeol in the total lipids, in core lipids, and in HPH-, MH-, DH-, and PH-GDGT fractions were performed (Figure 3). Similar to the NMDS results from AASV analyses, samples within and below DCM were clustered together and were well separated from samples above the DCM, but grouped together less tightly (Figure 2B).

A non-targeted full scan analysis was also applied here, as well as targeted screening method for the derivatives of GDGTs and archeol including unsaturated, hydroxy and methoxy counterparts as reported in Elling et al. (2014, 2017), with one exception for unsaturated archeol which was not targeted. This lipid class was not identified in our samples from full scan method. And none of these derivatives or lipids related to the production from Archaea were detected in surface water. Only Glycerol Dibiphytanol Diethers (GDDs) were observed at and below 100 m in KM1709_S16 and within and below the DCM in HOT296_S2, which were reported to be components of membrane lipids in Thaumarchaeota (Elling et al., 2014, 2017).

### Detection and Analyses of the Putative Homologs of Archaeal GDGT Ring Synthases (GrsA and GrsB) in MGII/III Archaea, and Their Distributions With Depth

**FIGURE 4:**
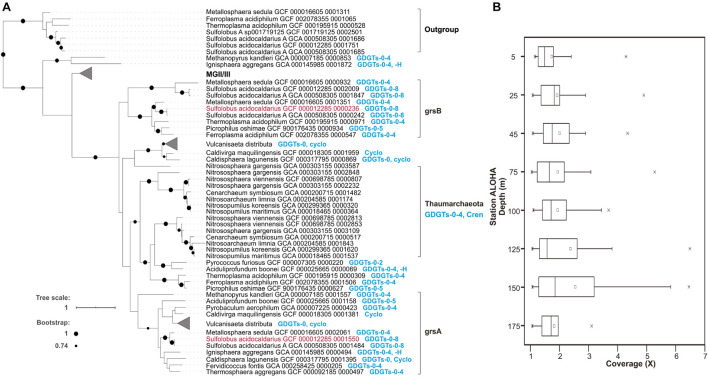
Putative Grs homologous proteins from MGII/III and their distributions in Station ALOHA. **(A)** The phylogenetic relationships of archaeal Grs homologs, and their correspondence to GDGT membrane compositions of known cultivars. The Grs homologs grsA and grsB whose function was experimentally verified in Zeng et al. (2019) are colored in red. The MGII/III Thermoplasmatota clade is shown in further detail in Supplementary Figure 16. **(B)** The metagenomic sequence read coverage profile of putative Grs homologs in the euphotic zone at Station ALOHA.

The radical S-adenosylmethionine (SAM) genes encoding GrsA and GrsB in *Sulfolobus acidocaldarius* were recently experimentally verified as having authentic archaeal GDGT ring synthase activity in this archaeal thermophile, and homologs were identified in Thaumarchaeota genomes (Zeng et al., 2019). We therefore used these genes to identify candidate homologous proteins from planktonic MGII/III Archaea. A total of 120 homologous sequences from MGII/III Archaea were detected in the GTDB database (Parks et al., 2018) and 52 representatives of putative proteins were in the Station ALOHA gene catalog, at the identity of 20–35% with Grs sequences from *S. acidocaldarius* (Figure 4, Supplementary Figure 16, and Supplementary Table 3). An *E*-value cutoff of ≤1e^–5^ was used here for retrieving homologs at low stringency, since mesophilic archaeal proteins may be only distantly related to (hyper)thermophilic GrsA and GrsB proteins identified in Zeng et al. (2019). Consistent with our results in Station ALOHA surface waters (data not shown), Zeng et al. (2019) reported the near absence of Thaumarchaeota GrsA/B homologs in surface waters in Station ALOHA metagenomes, with the vast majority of Thaumarchaeota GrsA/B homologs appearing within and below the DCM.

To further explore the phylogenetic relationships among GrsA and GrsB protein homologs, we constructed a rooted phylogenetic tree that included inferred protein sequences from MGI, II, and III and other archaeal isolates, as described in Supplementary Table 1 of Zeng et al. (2019) and retrieved from the GTDB database and ALOHA gene catalog. The phylogenetic tree showed that GrsA and GrsB clades consisted exclusively of putative homologs from thermophilic isolates that are known to produce cyclized GDGTs (Figure 4A). The putative homologs from MGI grouped together, and were only distantly related to archaeal thermophile GrsA and GrsB (Figure 4A). Compared to the outgroup of Saci_1785 radical SAM homologs, all of the putative MGII/III homologs clustered together as an ingroup, although they were more distantly related to clades of GrsA and GrsB from known and cultivated Archaea (Figure 4A and Supplementary Figure 16).

The metagenomic sequence read coverage of putative homologs from MGII/III was calculated for each sample from different depths within the euphotic zone at Station ALOHA collected during November 2014 to March 2015. The coverage values for each homolog per sample were lower above DCM, ranging between 1 and 5.22X coverage with an average of 1.86X, and were slightly elevated within and below the DCM, ranging between 1.01X and 6.43X coverage, with an average of 2.32X (Figure 4B). Since multiple GrsA/B copies have been found in a variety of Archaea (Zeng et al., 2019 and Figure 4 and Supplementary Table 3 in this study), we did not attempt to estimate the abundance of MGII/III Archaea based on the Grs homolog abundances.

### Calculation of Possible Cellular GDGTs and Archeol for Planktonic Thermoplasmatota

Quantitative estimates of Archaea, Thermoplasmatota, and Thaumarchaeota SSU rRNA gene abundances were obtained via ddPCR assays, using universal SSU rRNA primers, in combination with microbial community structure analyses achieved by high-throughput sequencing of the same PCR products. The absolute abundance of Thaumarchaeota changed markedly in depth profiles (ranging between 0 and 6.42 × 10^6^ SSU rRNA copies/L), whereas total archaeal SSU rRNA genes (ranging between 2.11 × 10^6^ and 1.22 × 10^7^ SSU rRNA copies/L) and total Thermoplasmatota SSU rRNA genes (ranging between 2.11 × 10^6^ and 8.55 × 10^6^ SSU rRNA copies/L) varied much less with depth (Figure 1 and Supplementary Table 1).

**FIGURE 5:**
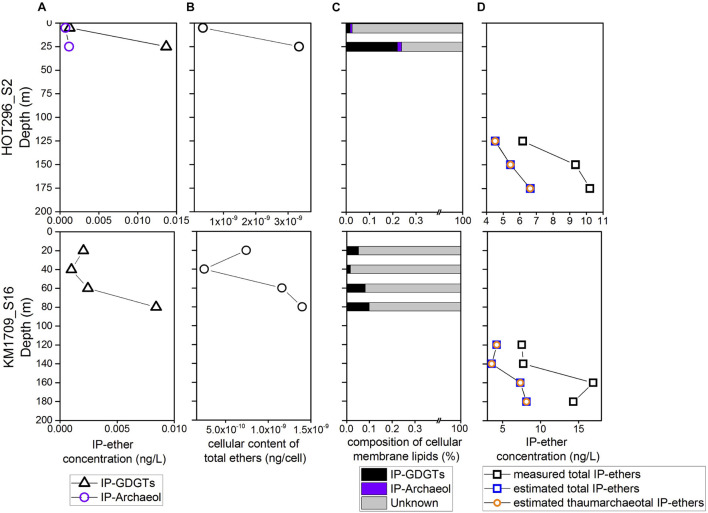
The estimates of cellular content of GDGTs and archeol from Thermoplasmatota and their contribution to the intact polar lipid pool. **(A)** The measured abundances of total IP-GDGTs at the surface. **(B)** The cellular content of GDGTs and archeol estimated for Thermoplasmatota. **(C)** The relative abundance of GDGTs and archeol in cellular membrane lipids of Thermoplasmatota. **(D)** The comparison of measured and estimated intact polar ether lipids within and below DCM, showing the latter IP-ethers are dominantly from Thaumarchaeota.

At depths shallower than 45 m at HOT296_S2 and 100 m at KM1709_S16, only Thermoplasmatota rRNAs were detectable, and their abundances ranged from 2.11 × 10^6^ to 6.02 × 10^6^ SSU rRNA copies/L (Figure 1 and Supplementary Table 1). Within these depths, concentrations of total IP-ethers ranged from 0.0010 to 0.015 ng/L (Figure 5A and Supplementary Table 1). We estimated the potential cellular ether lipid content of Thermoplasmatota, assuming they were the origin of the co-occurring ether lipids. The large majority of marine planktonic Bacteria and Archaea are considered to contain one to two copies of SSU rRNA gene per cell (Brown and Fuhrman, 2005; Needham et al., 2018), which may be their ecological strategies to grow in the oligotrophic environments (Klappenbach et al., 2000). Pure cultures of marine Thaumarchaeota, *Nitrosopumilus maritimus*, contain only one copy in the genome (Spang et al., 2010). Here, one copy was used for Thermoplasmatota and Thaumarchaeota. Using the ratio of total IP-ethers to total SSU rRNA gene abundance of Thermoplasmatota, the lipid content was 2.5 × 10^–10^ to 3.34 × 10^–9^ ng/cell, with an average of 1.21 × 10^–9^ ng/cell (Figure 5B and Supplementary Table 1). Lipids in Archaea with a cell diameter of 500 nm are estimated to be 1.4 × 10^–6^ ng/cell (Lipp et al., 2008). Based on this assumption, GDGTs appeared to comprise only 0.017–0.22% of the expected intact polar lipid content per Thermoplasmatota cell, while archeol constituted 0–0.019% (Figure 5C and Supplementary Table 1).

To estimate the abundance of IP-ethers derived from the biomass of MGII/III Thermoplasmatota and MGI Thaumarchaeota within and below DCM in comparison with the measured counter parts, we used the conversion factor for Thermoplasmatota ethers in the upper water column (1.21 × 10^–9^ ng/cell), and the published value for Thaumarchaeota ethers (1.27 × 10^–6^ ng/cell; Elling et al., 2014). Halobacterota probably contributing to the intact polar lipid pool within and below the DCM, only constituted less than 1% of archaeal populations, and thus their producing IP-ethers were not considered here. Within and below DCM, the estimated IP-ethers derived from MGII/III Archaeal biomass ranged between 5.18 × 10^–3^ and 1.03 × 10^–2^ ng/L, and estimated thaumarchaeotal IP-ethers were between 3.57 and 8.15 ng/L (Figure 5D and Supplementary Table 1). Hence, less than 1% of estimated total IP-GDGTs/ethers could be attributable to MGII/III Archaea within and below the DCM if Thermoplasmatota are in fact a source of these ether lipids. Within the same depths, the estimated abundances of total IP-ethers from all sources varied from 4.53–6.64 ng/L in HOT296_S2, and from 3.57 to 8.16 ng/L in KM1709_S16; whereas the measured total IP-ethers ranged between 6.18 and 10.21 ng/L in HOT296_S2 and between 7.54 and 16.92 ng/L in KM1709_S16 (Figure 5D and Supplementary Table 1). The measured concentrations of the total IP-ethers were about two to three-fold greater than expected. Here, we estimated the cellular content of ether lipids for Thaumarchaeota based on the concentration of IP-ethers relative to the SSU rRNA gene abundance of Thaumarchaeota detected within and below the DCM. This value ranged between 1.74 × 10^–6^ and 2.93 × 10^–6^ ng/cell with an average of 2.31 × 10^–6^ ng/cell.

## Discussion

### The Near-Exclusive Presence of Thermoplasmatota in Oligotrophic Surface Waters

The NPSG occupies ∼40% of the Earth’s surface and represents the largest circulation feature on Earth (Karl and Church, 2014), characterized by near-permanent stratification and low seasonality (Bingham and Lukas, 1996; Bryant et al., 2016; Karl and Church, 2017). The general characteristics of the NPSG generate strong depth structuring of microbial community composition in the water column, with most near-surface variability attributable to seasonal changes in day-length and light, and sporadic wind speed variation (DeLong et al., 2006; Bryant et al., 2016).

To better understand the ecology and biogeochemistry of planktonic archaeal populations, we performed synoptic measurements of their abundance patterns and membrane ether lipid distributions in the epipelagic zone of the NPSG. Planktonic Thermoplasmatota and planktonic Thaumarchaeota were confirmed as the two main groups of Archaea in the water column. Thermoplasmatota were abundant at all depths, while Thaumarchaeota increased in abundance within and just below the DCM. These observations are generally consistent with previous reports in the NPSG (DeLong et al., 2006; Brown et al., 2009; Church et al., 2010) and other oceanic regions (Massana et al., 1997, 2000; Mincer et al., 2007; Hugoni et al., 2013; Parada and Fuhrman, 2017).

The archaeal communities observed within the samples collected tended to separate into two general clusters, one above the DCM and one within and below the DCM. Thermoplasmatota (MGII and MGIII) dominated above the DCM, while within and below the DCM both Thermoplasmatota and MGI Thaumarchaeota were prevalent. Thaumarchaeota were not detected at depths shallower than 45 m at HOT296_S2 and 100 m at KM1709_S16 (Figure 1), again consistent with previous reports in this region (Brown et al., 2009). Based on Station ALOHA gene catalog data, the analysis of ribosomal protein S3 (RpS3) showed no presence of MGI Thaumarchaeota from samples shallower than 45 m during November 2014 to April 2016 (Supplementary Table 4). One reason may be the inhibitory influences of sunlight on growth of nitrifying Thaumarchaeota in surface waters (Church et al., 2010; Merbt et al., 2012; Horak et al., 2018).

The universal SSU rRNA gene PCR primers used here have been reported to remove biases against Thaumarchaeota (Parada et al., 2016). Based on ddPCR and sequencing using this universal primer set, MGI Thaumarchaeota abundances were approximately 10^6^ SSU rRNA copies/L within and below DCM (Figure 1 and Supplementary Table 1), similar to amoA gene densities reported at these depths in the same oceanic region (Church et al., 2010). These observations were also supported using an archaeal-specific primer set (ARC344FB/ARC519R; Raskin et al., 1994; Sørensen and Teske, 2006; Teske and Sørensen, 2008). These archaeal-specific primers also showed the complete absence of Thaumarchaeota in NPSG surface waters (Supplementary Figure 17). In general, total archaeal SSU rRNA amplicon-based richness and Shannon indices were much lower above the DCM in both stations (Supplementary Figure 4), compared to those within and below the DCM. The rarefaction curves also reached an asymptote (Supplementary Figure 3).

The MGIII planktonic Archaea detected in this study accounted for an average of approximately 30% of total archaeal sequences recovered, with a maximum abundance of 48.1% at 60 m of KM1709_S16 (Figure 1). While few studies have reported the presence of MGIII Archaea in the euphotic zone (Brown et al., 2009; Galand et al., 2010; Haro-Moreno et al., 2017), they represented up to 10% of all archaeal sequences in samples from the Mediterranean DCM (Galand et al., 2010), and 11.8% at 10 m depth at station ALOHA from HOT cruise 169 (Brown et al., 2009). Haro-Moreno et al. (2017) reconstructed epipelagic MGIII genomes by binning the assembled fragments, which contained numerous photolyase and rhodopsin genes as well as genes coding for degradation and uptake of protein and lipids. These metabolic features seem complementary to oligotrophic conditions found in surface waters, and may allow MGIII with photoheterotrophic lifestyles to thrive, like their MGII relatives, in the ocean’s euphotic zone (Brown et al., 2009; Galand et al., 2010; Haro-Moreno et al., 2017). MGIII Archaea therefore appear to have the potential to live in the euphotic zone, and they may at times be comparable in abundance to MGII Archaea.

### Planktonic Thermoplasmatota Contributions to Surface Water GDGTs

From larger volumes of seawater (>3000 L/sample), Hurley et al. (2018) reported HPH-, PH-, DH-, MH-, and C-GDGT distribution patterns along depth profiles between 0 and 1000 m. The total lipid concentration reported by Hurley et al. (2018) exhibited a similar pattern to those found here with low concentrations detected above the DCM (minimum 0.0015 ng/L at 5 m) and high concentrations within and below the DCM (maximum 11.84 ng/L at 158 m) (Supplementary Figure 18). Meanwhile, concentrations of the total intact polar lipids also showed the minimum (0.00021 ng/L) and maximum (8.94 ng/L) values at these same depths (Hurley et al., 2018). GDGT compositions within and below the DCM in our study here resembled those of Hurley et al. (2018) whereas above the DCM, similarities were seen mainly in HPH- and MH-GDGTs from HOT296_S2, and in DH- and MH-GDGTs in KM1709_S16 (Supplementary Figure 18). Compared to the study in Hurley et al. (2018), the depth profiles of GDGTs in our study showed a larger difference, likely due to the oligotrophic condition and near-permanent stratification in our stations. We used NMDS analysis to decipher the relationship of the GDGT distribution among the different samples reported by Hurley et al. (2018) and found the same clustering patterns as we report here, except that all their samples at Station 2 grouped together (Supplementary Figure 19). In depth profiles of the relative distributions of different GDGT classes (see the last plot for each station in Supplementary Figure 18), C-GDGTs were much more abundant above, than within and below the DCM in Station 2, which was also observed for their Stations 7, 15, and 23 (Hurley et al., 2018).

A Mantel test was conducted to explore the correlation between the compositions of the archaeal community (based on AASVs) and ether lipid distribution (based on all lipids) by Bray-Curtis distance, with 999 permutations. The significant relationship identified (significance = 0.001) suggests that the biosynthesis of GDGTs and archeol is attributable to specific archaeal populations, as reported in previous studies (Pearson and Ingalls, 2013; Schouten et al., 2013; Lincoln et al., 2014; Sollai et al., 2018). MGII and MGIII planktonic Archaea are therefore still potentially a source of these lipids in shallow open ocean waters, consistent with the findings of Ma et al. (2020) in Northwestern Pacific Ocean surface water. The different depth profiles of GDGTs observed in the surface water between two stations are probably due to the divergent phylogenies in Thermoplasmatota (Rinke et al., 2019), which could also be seen in the distant relationships of archaeal ASVs detected at these two sites (Figure 2). Our results contrast with a recent report by Besseling et al. (2020) in surface waters of the coastal North Sea and the North Atlantic Ocean, where MGII/III Thermoplasmatota dominated. However, the threshold of detection for archaeal IP-GDGTs using their analytical methods was reported to be about 7 × 10^6^ Thaumarchaeota SSU rRNA copies/L (Besseling et al., 2020. In our study here, IP-GDGTs were detected in surface waters where MGI Thaumarchaeota were absent and MGII/III Thermoplasmatota were very abundant. The potential for Thermoplasmatota tetraether lipid production was also supported by the presence of genes encoding enzymes associated with ether bond formation and isoprenoid saturation in MAGs of representatives of these two archaeal groups (Iverson et al., 2012; Deschamps et al., 2014; Villanueva et al., 2017). While a small SSU rRNA signal was detected from Woesearchaeota in surface waters on the HOT296_S2 cruise (Figure 1), no ether bond formation genes have yet been reported in MAGs from this group of Archaea (Villanueva et al., 2017).

### Detection of GDGT Ring Synthase Homologs in Planktonic Thermoplasmatota

Zeng et al. (2019) recently identified two GDGT ring synthases in *S. acidocaldarius*, GrsA and GrsB (having similarity of 31%), which were found to introduce rings separately at the C-7 and C-3 positions of the core GDGT lipids, with GrsB preferring substrates with existing C-7 rings. Phylogenetic analysis indicates that GrsA and GrsB clades are found predominantly in (hyper)thermophiles, with a few sequences from mesophilic Archaea that mostly belonged to Bathyarchaeota and Thermoplasmatales, and many of them contain the two paralogs (Zeng et al., 2019). The putative homologs from planktonic Thaumarchaeota clustered into a group separate from (hyper)thermophilic archaeal GrsA and GrsB clades (Zeng et al., 2019). Given that Thaumarchaeota are phylogenetically affiliated with the Crenarchaeota, it is perhaps not surprising that their Grs homologs share significant protein sequence identity, even though these genes originate from mesophiles or psychrophiles.

Our phylogenetic observations are consistent with those of Zeng et al. (2019), and we report here additionally that GrsA homologs are also found in planktonic Thermoplasmatota (Figure 4B, Supplementary Figure 16, and Supplementary Table 3). It is possible that since MGII/III Thermoplasmatota are mesophilic organisms in ocean surface waters, they do not produce multiple cyclopentane rings (i.e., five cyclopentane rings or more) that are characteristic of membrane lipids in acidophiles, halophiles, and thermophiles (Pearson and Ingalls, 2013; Schouten et al., 2013). The planktonic Thermoplasmatota ring synthase homologs belonged to the Grs clade, and were more closely related to GrsA and GrsB clades, than some other archaeal homologs, for example those from extremely thermophilic isolates *Methanopyrus kandleri* and *Ignisphaera aggregans* (also known to produce cyclized GDGTs) which although have not been tested yet for the same function as GrsA and GrsB (Figure 4A). The catalytic properties of mesophilic MGII/III Thermoplasmatota ring synthase may be distinct from GrsA and GrsB of (hyper)thermophiles identified in Zeng et al. (2019), since their candidate homologs exhibit only distant relationships to GrsA and GrsB families. Future studies need to put more focus on the distribution and function of GDGT synthases from mesophiles. At present however, we cannot exclude the possibility that the putative MGII/III Thermoplasmatota homologs identified here are other radical SAM proteins.

### Cellular Content of Ether Lipids in Planktonic Thermoplasmatota

While we assumed that trace MGI Thaumarchaeota would exist and be contributor to the IP-ether pools within depths shallower than 45 m at HOT296_S2 and 100 m at KM1709_S16, the surface MGI thaumarchaeotal abundance estimated would range from 7.87 × 10^2^ to 1.17 × 10^4^ SSU rRNA copies/L with an average of 4.03 × 10^3^ SSU rRNA copies/L, based on concentrations of the total IP-ethers measured and the thaumarchaeotal cellular content (1.27 × 10^–6^ ng/cell, Elling et al., 2014) of GDGTs and archeol (Supplementary Table 1), Therefore, the estimated abundance of Thaumarchaeota relative to the total archaeal biomass detected would vary between 0.020 and 0.26% (a mean value of 0.10%), which exceeds the theoretical detection limit of our method (approximately 0.021–0.059% with a mean value of 0.038%) using the universal PCR primers. Similarly, the archaeal-specific primer set yielded 90,104–127,794 high-quality reads per sample (a mean value of 107,817) at the surface, with a detection limit of approximately 0.00078–0.0011% (a mean value of 0.00094%). Therefore, if Thaumarchaeota SSU rRNA genes were present in surface waters, they should have been detectable, yet they were not.

We also calculated the cellular content of ether lipids in planktonic Thermoplasmatota inhabiting the surface or MGI Thaumarchaeota within and below the DCM, assuming the ether lipids originate from these groups of Archaea. The average value of 1.21 × 10^–9^ ng/cell for Thermoplasmatota in surface waters, was three orders of magnitude lower than reported cellular ether content for cultivated MGI Thaumarchaeota (1.27 × 10^–6^ ng/cell, Elling et al., 2014). Subsurface MGI Thaumarchaeota had a mean lipid content of 2.31 × 10^–6^ ng/cell, two fold higher than the culture estimate, suggesting the need to derive more accurate lipid/cell conversation factors in future studies. Another explanation would be that cell diameters of Thaumarchaeota within and below the DCM in Station ALOHA may be larger than the cultivated one in Elling et al. (2014). The difference in the analytical procedures used in our study and in Elling et al. (2014) may also be responsible.

### Ecological and Geological Implications

There are several potential explanations for the combined absence of Thaumarchaeota rRNA-genes in surface waters, along with low estimated cellular IP-ether lipids in MGII/III Thermoplasmatota found in the same habitat. It is possible that small numbers of Thaumarchaeota cells may be transported upward, either by mixing of thaumarchaeotal cells into surface waters, by vertical migrators or buoyant transport from greater depths (Yayanos and Nevenzel, 1978; Smith et al., 1989; Sommer et al., 2017). In inhospitable surface waters, upwardly transported thaumarchaeotal cells may not survive, and their nucleic acids might decay more rapidly than their lipids. This could explain the absence of thaumarchaeotal SSU rRNA genes in surface waters, while small traces of their lipids remain. A related scenario invokes surface water GDGTs as thaumarchaeotal membranes or micelles, upwardly transported in similar ways. Cultured MGI Thaumarchaeota have been reported to synthesize crenarchaeol as their major membrane lipid with lower abundances of GDGTs-0 to 3 (Sinninghe Damsté et al., 2002; Elling et al., 2014, 2015, 2017), consistent with the GDGT distributions within and below the DCM in our samples. At 5 and 25 m in HOT296_S2, crenarchaeol was determined to be 12-fold greater than GDGT-2 and at least 23-fold greater than the remaining GDGTs (calculated based on the estimated quantification limit of 0.00060 ng/L); whereas GDGT-0 was at least 6- or 9-fold greater than crenarchaeol at 20 and 40 m in KM1709_S16 (Figure 3 and Supplementary Table 1). The GDGT profiles observed in the surface water samples are quite distinct from those produced by cultured MGI Thaumarchaeota however, so potential explanations invoking upward transport of Thaumarchaeota cells or lipids to the surface waters seem unlikely, or would have to include a mechanism for selectively transporting a structural subpopulation of the lipid pool.

The possibility still remains then, that planktonic Thermoplasmatota were the sole source of surface water GDGTs we report here in the NPSG samples. The facts that planktonic Thermoplasmatota were the only detectable Archaea in surface waters, and that their genomes encoded ether lipid biosynthetic gene homologs (Iverson et al., 2012; Deschamps et al., 2014; Villanueva et al., 2017) as well as GrsA homologs (this study), support this hypothesis.

If planktonic Thermoplasmatota were the source of surface water GDGTs and archeol, why did these lipids comprise less than an estimated 0.3% of their cellular lipids (Figure 5C)? This could be explained by the presence or predominance of lipids other than GDGTs or archeol that were not measured in our study, in keeping with a recent study of Besseling et al. (2020). It is possible that other common archaeal lipids such as other phytanyl ether lipids comprise the dominant components of MGII and MGIII planktonic archaeal membrane lipids (Villanueva et al., 2017).

With respect to implications for archaeal lipid export flux, the surface ocean is characterized by higher particle density and enhanced rates of protistan grazing and mesoplankton packaging, which could increase the incorporation of prokaryotic biomass into sinking flux (Richardson and Jackson, 2007; Close et al., 2013). The particle sinking rates reported can be as great as 820 m/d at Station ALOHA (Trull et al., 2008). Phytoplankton in the surface ocean have rapid growth and short generation times. Under these conditions, the contribution of surface MGII/III to subsurface and deeper water GDGTs could not be ignored. This may be part of reason that the estimated cellular content of ether lipids in Thaumarchaeota within and below the DCM is two-fold higher than the corresponding culture estimates. The biological origins of GDGTs exported to sediments have to be interpreted with caution. If MGII/III Thermoplasmatota are the source of surface water GDGTs, this implies that they are also a source of crenarchaeol, challenging the assumption that crenarchaeol is a biomarker of Thaumarchaeota, which until recently were thought to be exclusively ammonia oxidizing chemoautotrophs (Aylward and Santoro, 2020). If this was in fact true, our understanding of nitrogen cycling processes or the activity of Thaumarchaeota reconstructed using crenarchaeol in paleo studies (Dong et al., 2019; Elling et al., 2019), may be more complex than current models would suggest. Future efforts, including the investigation of Thermoplasmatota growth and lipid content, as well as processes such as grazing, packaging, and export from surface waters, may better constrain the biological origins of sedimentary archaeal lipids. The enrichment and isolation of representatives of MGII/III Thermoplasmatota as well as approaches like BONCAT (Dieterich et al., 2006), isotope labeling experiments and natural carbon isotope ratio detection have potential to further improve our understanding of ecological and physiological functions of planktonic archaeal groups.

## Data Availability Statement

The datasets presented in this study can be found in online repositories. The names of the repository/repositories and accession number(s) can be found in the article/Supplementary Material.

## Author Contributions

ED and AI designed the work. FL and LC conducted the lipid experiments and collected the lipid data. FL conducted the DNA experiment, collected the DNA data, and analyzed the lipid data. FL and KP analyzed the amplicon sequence data. AL analyzed the metagenomic data. FL and ED wrote the manuscript. All authors contributed to the article and approved the submitted version.

## Conflict of Interest

The authors declare that the research was conducted in the absence of any commercial or financial relationships that could be construed as a potential conflict of interest.

## Publisher’s Note

All claims expressed in this article are solely those of the authors and do not necessarily represent those of their affiliated organizations, or those of the publisher, the editors and the reviewers. Any product that may be evaluated in this article, or claim that may be made by its manufacturer, is not guaranteed or endorsed by the publisher.

## Footnotes

1
https://github.com/scapella/trimal

2
http://itol.embl.de/

3
http://gtdb.ecogenomic.org/tree

## Supplementary Material

Supplementary Figure 1 Structures of core and intact polar GDGTs. Crenarchaeol isomer has a different stereochemistry in one of cyclopentane rings, compared to crenarchaeol (Sinninghe Damsté et al., 2018).

Supplementary Figure 2 Sampling locations and associated hydrographic data. Hydrographic data is displayed along depth profiles with an interval of 1 m for HOT206_S2, and similarly for KM1709_S16.

Supplementary Figure 3 Rarefaction curves of rRNA gene recoveries. The collectors curve derived for each sample was based on PCR reactions using universal primers **(A)**; or archaeal specific primers **(B)**. Archaeal specific primers, ARC344FB (5′-ACGGGGCGCAGCAGGCGCGA-3′, Raskin et al., 1994) and ARC519R (5′-GGTDTTACCGCGGCKGCTG-3′, Sørensen and Teske, 2006), were used to target the V3 region of the SSU rRNA gene.

Supplementary Figure 4 Archaeal community diversity and richness. Community metrics were calculated as richness **(A)** and Shannon index **(B)** along depths in both stations.

Supplementary Figure 5 Fragmentation patterns of protonated and ammoniated acid-hydrolyzed C-GDGT archaeal lipid fractions for the 140 m depth sample in KM1709_S16 and the extracted ion chromatograms for the 45 m depth sample in HOT296_S2. LC-MS chromatograms show distributions of and MS/MS spectra show characteristic fragment ions of protonated **(A)** and ammoniated **(B)** C-GDGTs from acid-hydrolyzed fraction of sample at 140 m of KM1709_S16. The retention time for each C-GDGTs is also shown in panel **(A)**. **(C)** Enlarged MS/MS spectra of protonated C-crenarchaeol from panel **(A)**. **(D)** The extracted LC-MS chromatograms of C-GDGTs from both non-hydrolyzed and acid-hydrolyzed fractions of sample at 45 m of HOT296_S2. Three compound peaks are labeled here for the potential assignment to GDGTs. The green colored one with m/z 1302 has the same retention time as protonated GDGT-0. The dark red colored one with m/z 1318 has the retention time very close to sodiated GDGT-3. The MS/MS spectra however, are not able to show the characterized fragmentation pattern of sodiated GDGT-3, which would be dominated by the precursor ion [M + Na]^+^ (m/z 1318), since this GDGT cannot be fragmented by our MS/MS method. The dark yellow colored one with m/z 1314 is eluted 1 min later than sodiated crenarchaeol.

Supplementary Figure 6 Fragmentation patterns of protonated and ammoniated acid-hydrolyzed archeol. LC-MS chromatograms show distributions of and MS/MS spectra show characteristic fragment ions of protonated and ammoniated archeol from acid-hydrolyzed fraction of sample at 120 m of KM1709_S16.

Supplementary Figure 7 Fragmentation patterns of protonated and ammoniated non-hydrolyzed HPH-GDGT archaeal lipid fractions. LC-MS chromatograms show distributions of and MS/MS spectra show characteristic fragment ions of protonated **(A)** and ammoniated **(B)** HPH-GDGTs from non-hydrolyzed fraction of sample at 175 m of HOT296_S2. **(C)** Enlarged MS/MS spectra of ammoniated HPH-GDGT-0 from panel **(B)**.

Supplementary Figure 8 Fragmentation patterns of protonated non-hydrolyzed PH-GDGT archaeal lipid fractions. **(A)** LC-MS chromatograms showing distributions of and MS/MS spectra showing characteristic fragment ions of protonated PH-GDGTs from non-hydrolyzed fraction of sample collected from a sediment trap at 4000 m in station ALOHA (Boeuf et al., 2019). **(B)** Enlarged MS/MS spectra of protonated PH-GDGT-0 from panel **(A)**. **(C)** LC-MS chromatograms and MS/MS spectra of protonated PH-GDGTs from non-hydrolyzed fraction of sample at 175 m of HOT296_S2.

Supplementary Figure 9 Fragmentation patterns of ammoniated DH-GDGT archaeal lipids. **(A)** LC-MS chromatograms showing distributions of and MS/MS spectra showing characteristic fragment ions of ammoniated DH-GDGTs from non-hydrolyzed fraction of sample at 175 m of HOT296_S2. **(B)** Enlarged MS/MS spectra of ammoniated DH-crenarchaeol from panel **(A)**.

Supplementary Figure 10 Fragmentation patterns ammoniated non-hydrolyzed MH-GDGT archaeal lipid fractions. **(A)** LC-MS chromatograms showing distributions of and MS/MS spectra showing characteristic fragment ions of ammoniated MH-GDGTs from non-hydrolyzed fraction of sample at 175 m of HOT296_S2. **(B)** Enlarged MS/MS spectra of ammoniated MH-crenarchaeol from panel **(A)**.

Supplementary Figure 11 Fragmentation patterns of C-GDGT and HPH-GDGT archaeal lipids from 5 m depth. LC-MS chromatograms showing distributions of and MS/MS spectra showing characteristic fragment ions of C-GDGTs **(A)** and HPH-GDGTs **(B)** on sample at 5 m of HOT296_S2.

Supplementary Figure 12 Fragmentation patterns of HPH-GDGT archaeal lipids from 25 m depth. LC-MS chromatograms showing distributions of and MS/MS spectra showing characteristic fragment ions of HPH-GDGTs on sample at 25 m of HOT296_S2.

Supplementary Figure 13 Fragmentation patterns of C-GDGT and HPH-GDGT archaeal lipids from 20 m depth. LC-MS chromatograms showing distributions of and MS/MS spectra showing characteristic fragment ions of C-GDGTs **(A)** and HPH-GDGTs **(B)** on sample at 20 m of KM1709_S16.

Supplementary Figure 14 Fragmentation patterns of C-GDGT, HPH-GDGT, and MH-GDGT archaeal lipids from 40 m depth. LC-MS chromatograms showing distributions of and MS/MS spectra showing characteristic fragment ions of C-GDGTs **(A)**, HPH-GDGTs **(B)** and MH-GDGTs **(C)** on sample at 40 m of KM1709_S16.

Supplementary Figure 15 Concentrations of individual GDGTs and archeol in total lipids from acid-hydrolyzed fraction along depths at both sampling stations.

Supplementary Figure 16 The cluster of Grs homologs originating from MGII/III as identified in Figure 4. Putative homologs from Station ALOHA are labeled in red.

Supplementary Figure 17 Abundance of specific archaeal groups as a function of depths. The relative distribution of archaeal community was estimated by amplicon sequencing of ddPCR products using archaeal specific primers.

Supplementary Figure 18 Concentrations of total GDGTs and relative abundances of GDGTs from Hurley et al. (2018). The sum of C-, HPH-, DH-, and MH-GDGTs, and C-, HPH-, DH-, MH-, and IP-GDGTs, as well as relative abundances of different classes of GDGTs at different depths as reported in data in Hurley et al. (2018). The DCM occurred at 50–75 m in Station 2, at 125 m in Station 7, at 60–70 m in Station 15, and at 70 m in Station 23.

Supplementary Figure 19 Non-metric multidimensional scaling plots of archaeal GDGT relative abundances from Hurley et al. (2018). Based on Bray-Curtis distance matrices generated from the relative abundances of archaeal GDGTs, as reported in data in Hurley et al. (2018). The DCM depths for each station were described in Supplementary Figure 18.

Supplementary Table 1 Sampling, hydrographic data, archaeal abundance estimates, estimated cellular ether lipid content for Thermoplasmatota, estimated and measured ether lipid concentrations.

Supplementary Table 2 High quality reads of the archaeal community.

Supplementary Table 3 The Grs homologs in MGII/III from the Genome Taxonomy Database and ALOHA gene catalog.

Supplementary Table 4 The count profile of Rps3 sequences from the ALOHA gene catalog at the surface of Station ALOHA.
