# Supplementary material for: Planktonic Archaeal Ether Lipid Origins in Surface Waters of the North Pacific Subtropical Gyre
Source: Front Microbiol. 2021 Sep 13;12:610675. doi: 10.3389/fmicb.2021.610675 (PMC8473941; doi:10.3389/fmicb.2021.610675)
Supplement: Supplementary Figure 1 — Structures of core and intact polar GDGTs. Crenarchaeol isomer has a different stereochemistry in one of cyclopentane rings, compared to crenarchaeol (Sinninghe Damsté et al., 2018). [file Presentation_1.pdf]

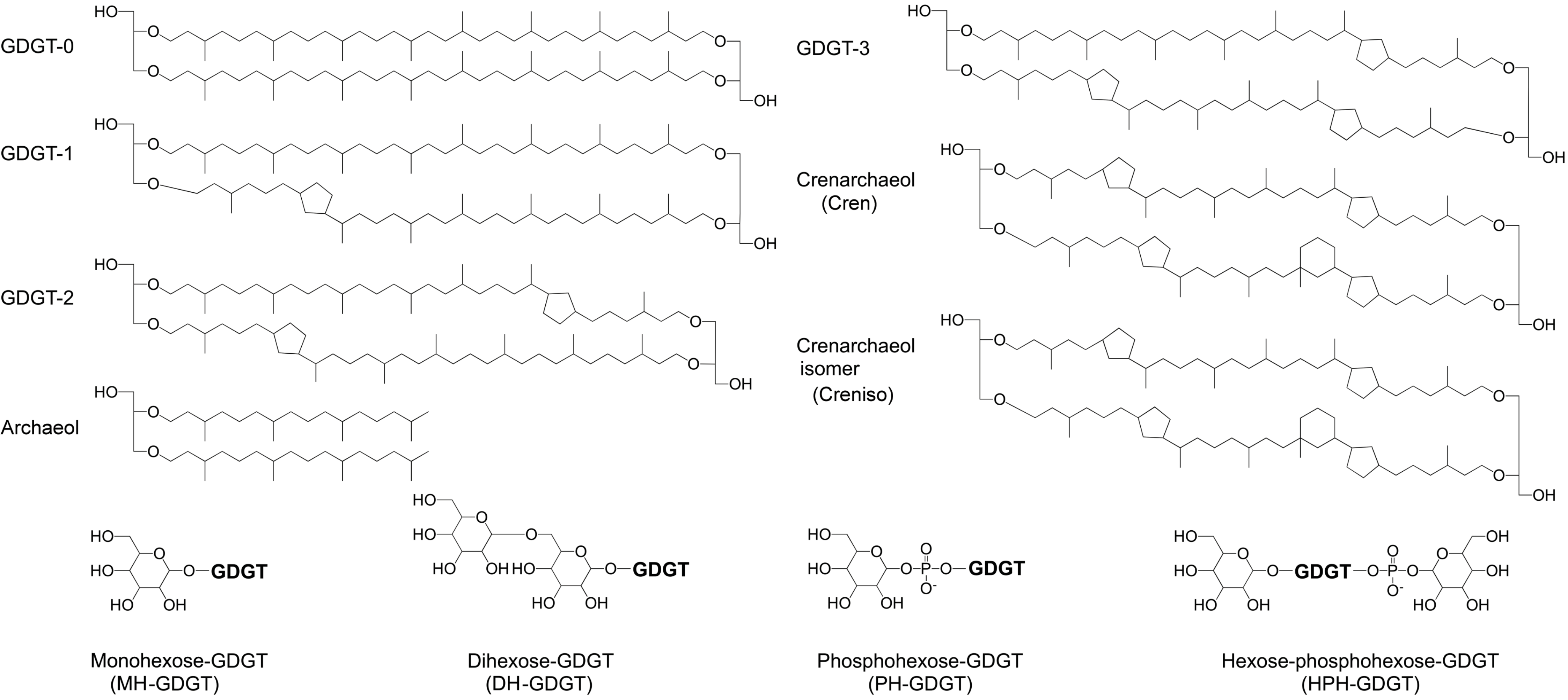

Supplementary Figure1. Structures of IP- and C-GDGTs

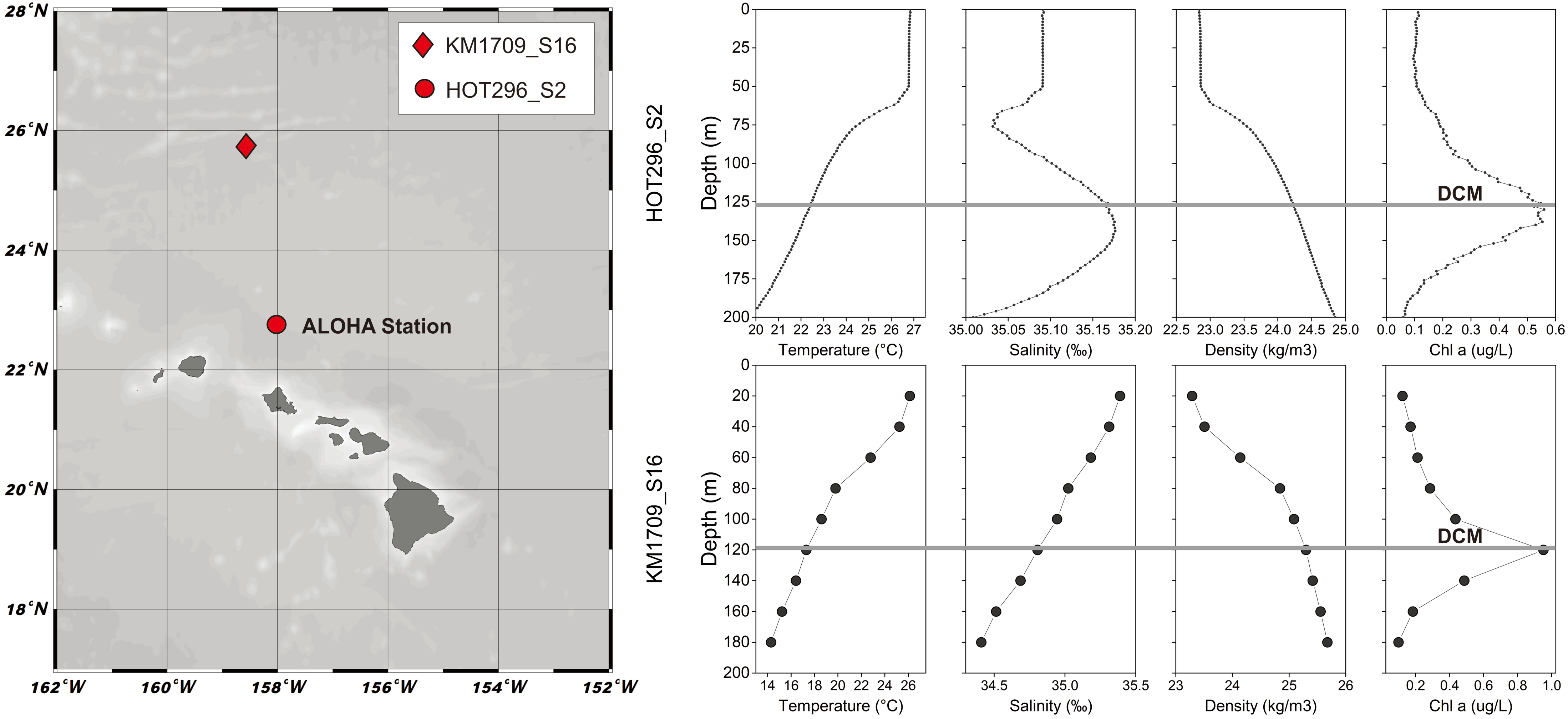

Supplementary Figure 2

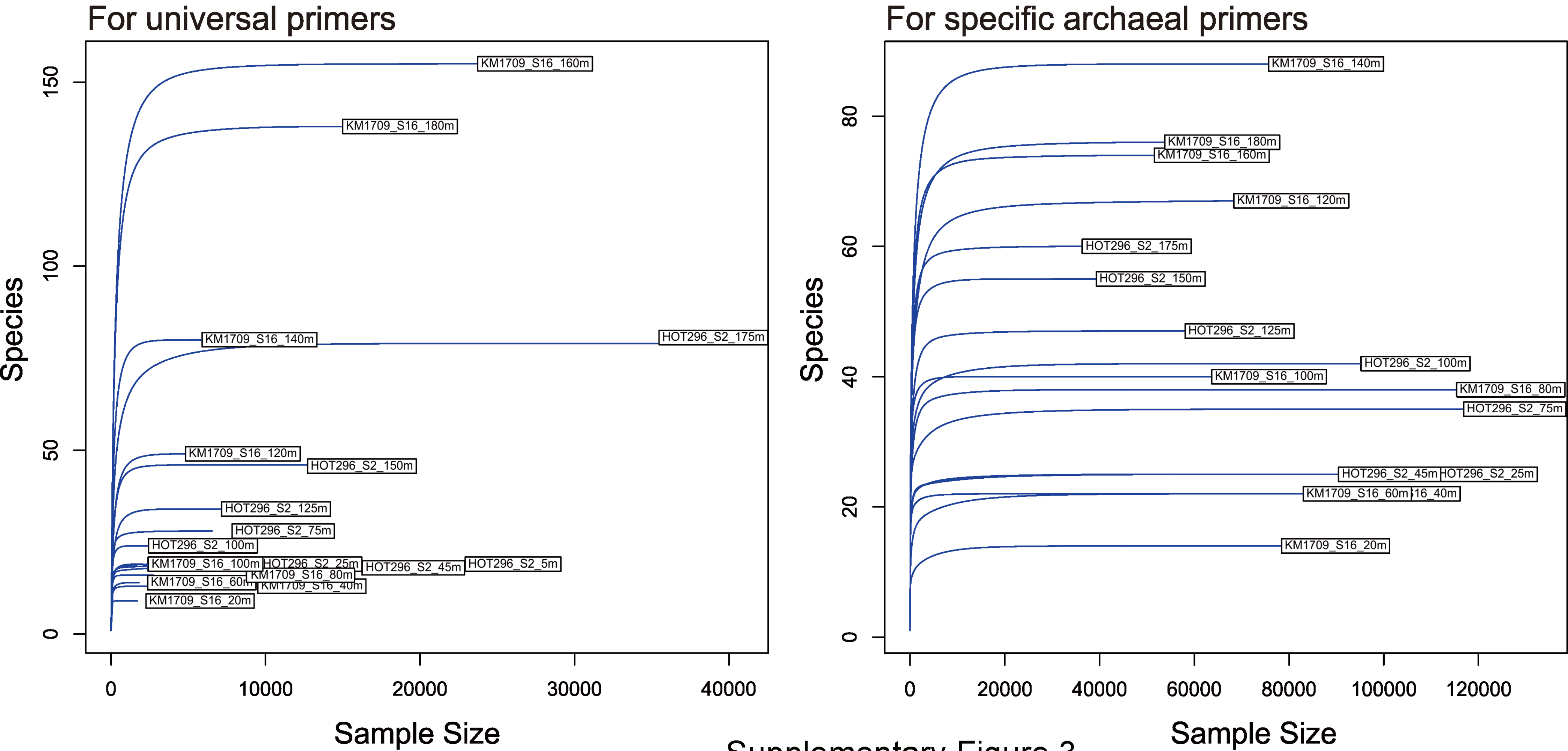

Supplementary Figure 3

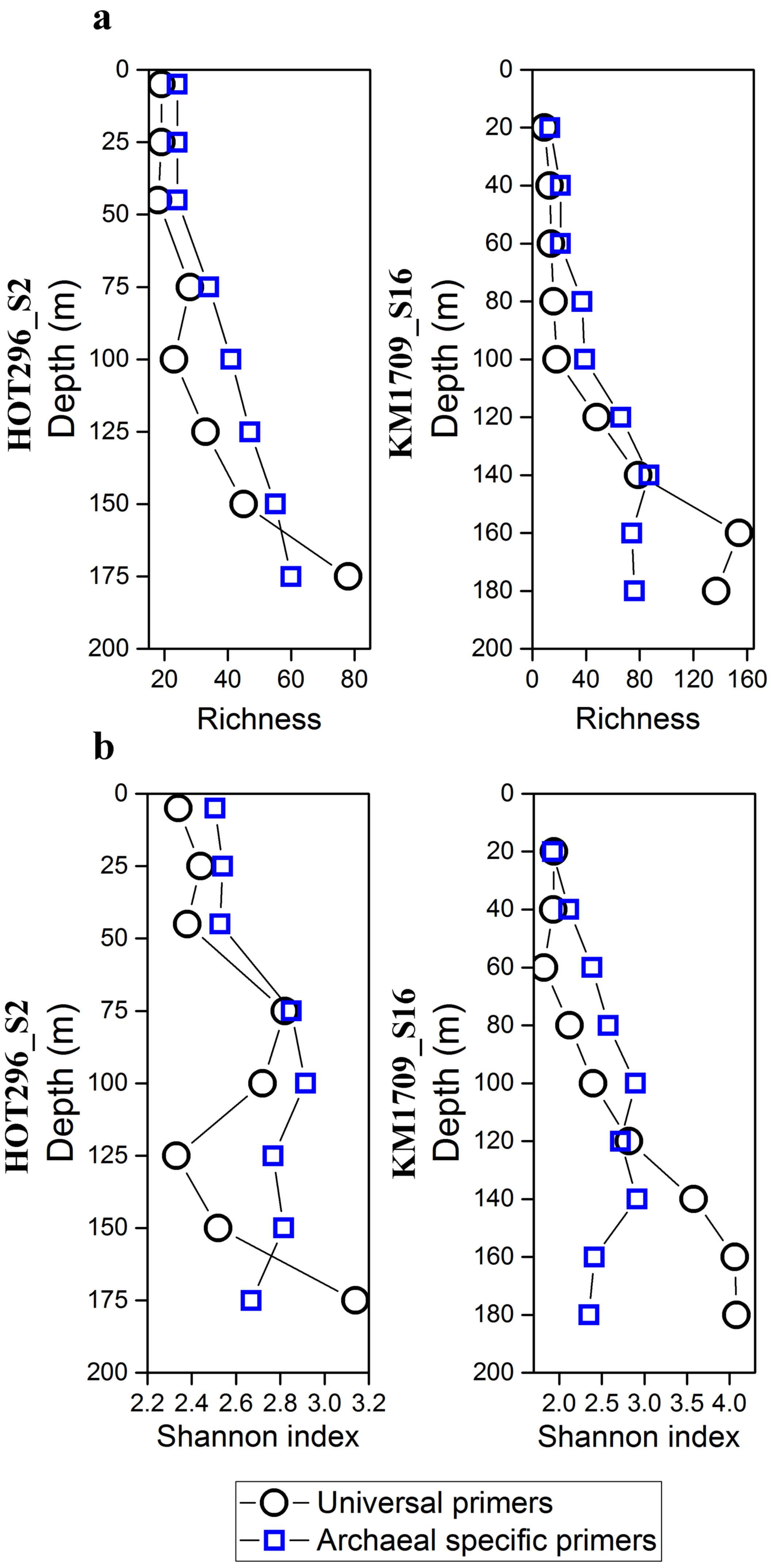

Supplementary Figure 4

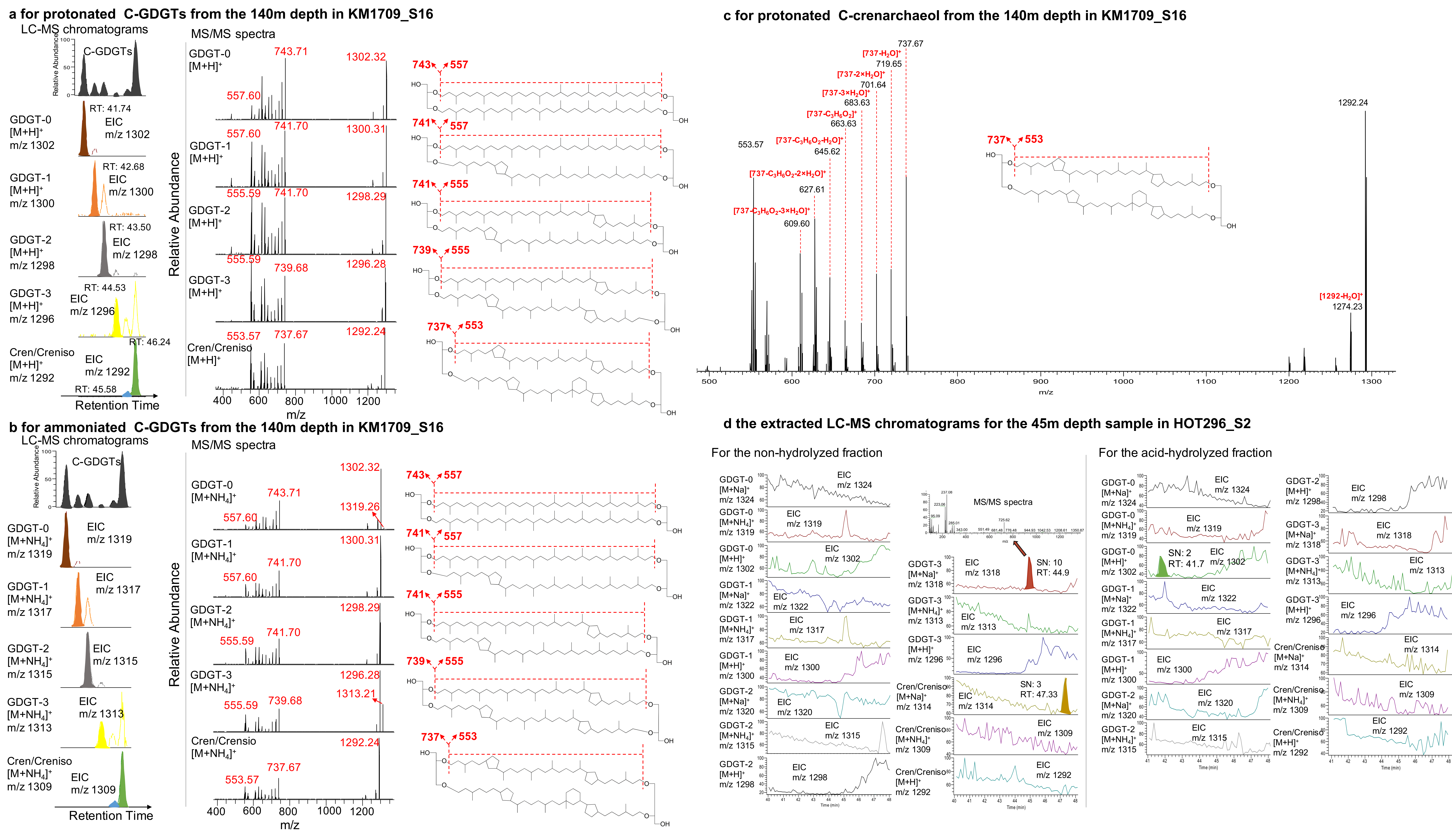

Supplementary Figure 5

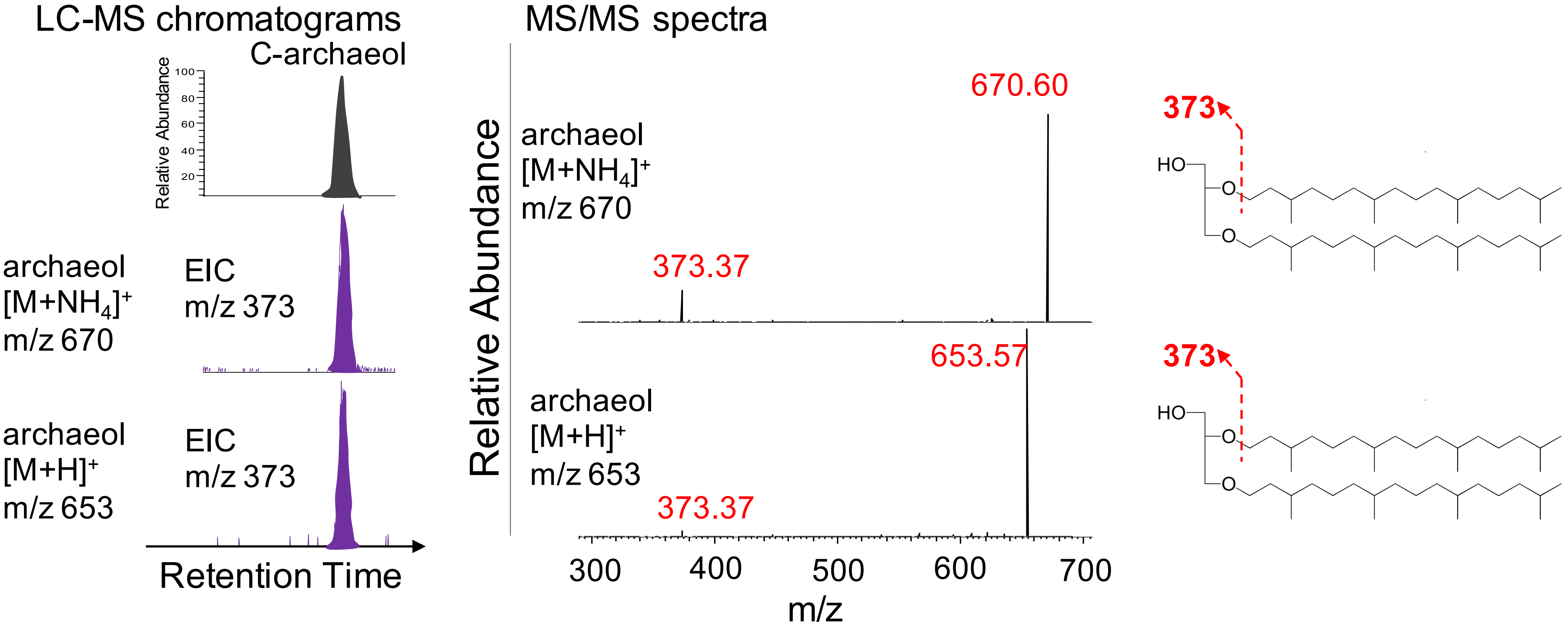

Supplementary Figure 6

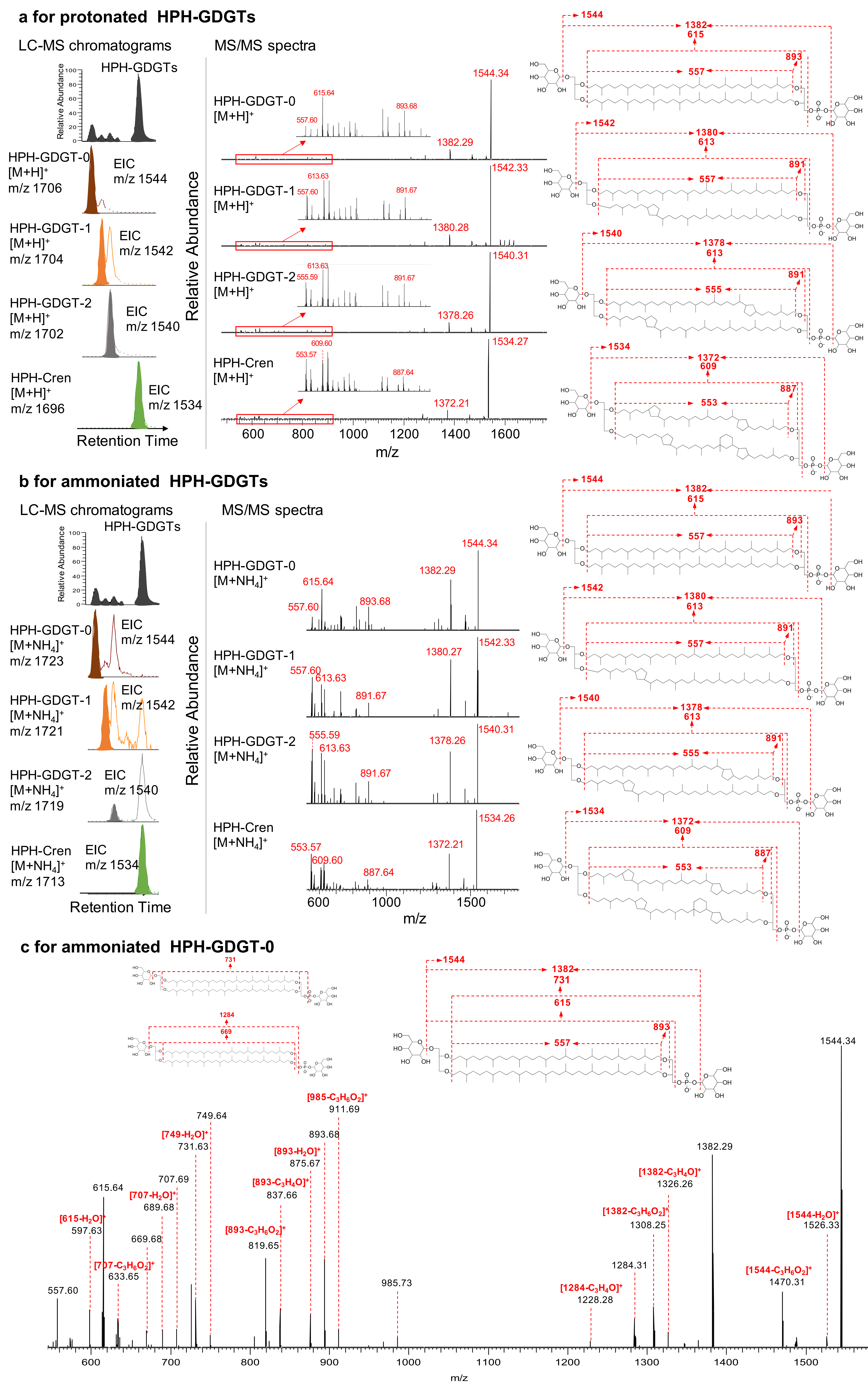

Supplementary Figure 7

a for protonated PH-GDGTs from sample of sediment trap at 4000m of station ALOHA

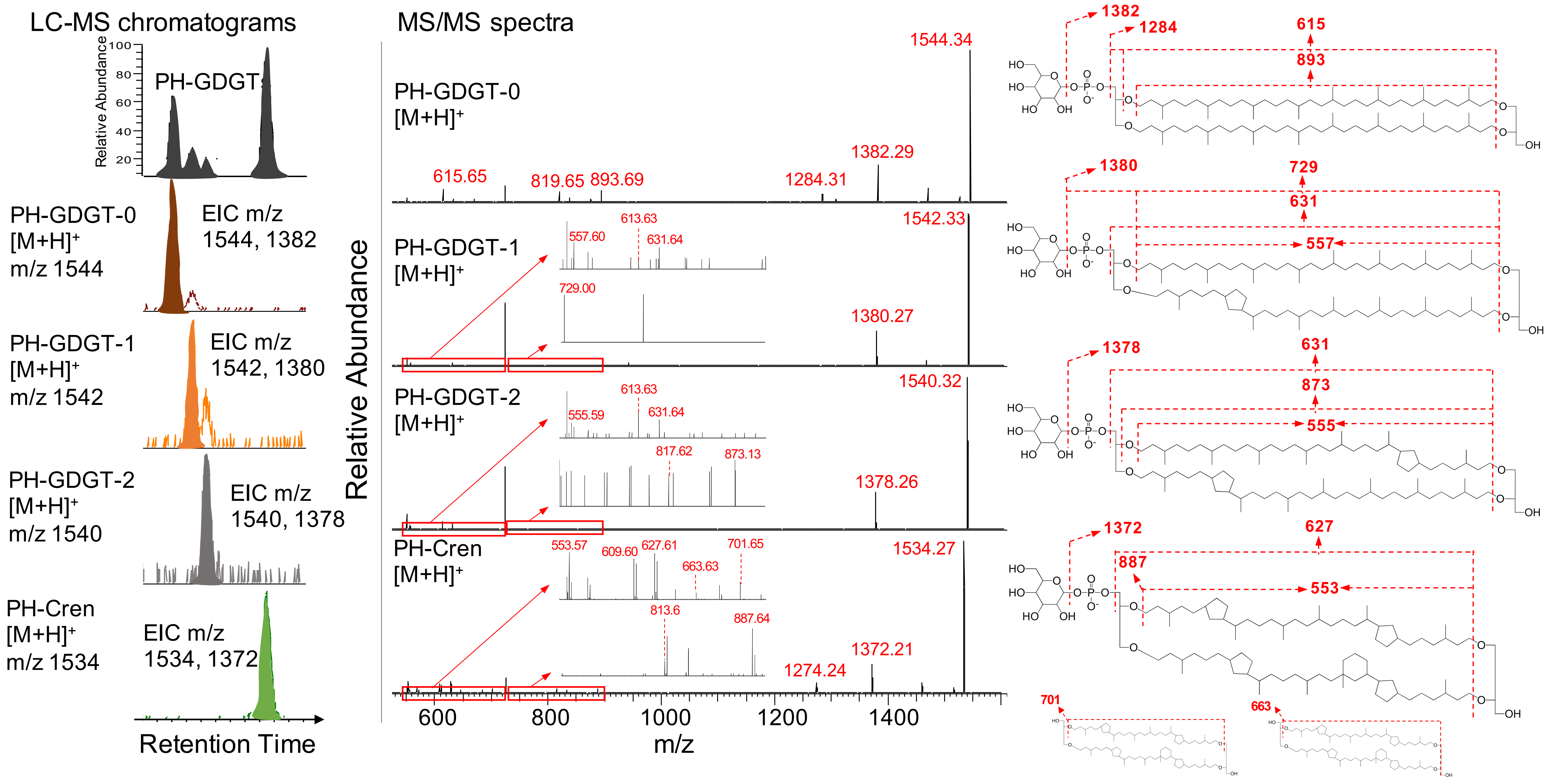

b for protonated PH-GDGT-0 from (a)

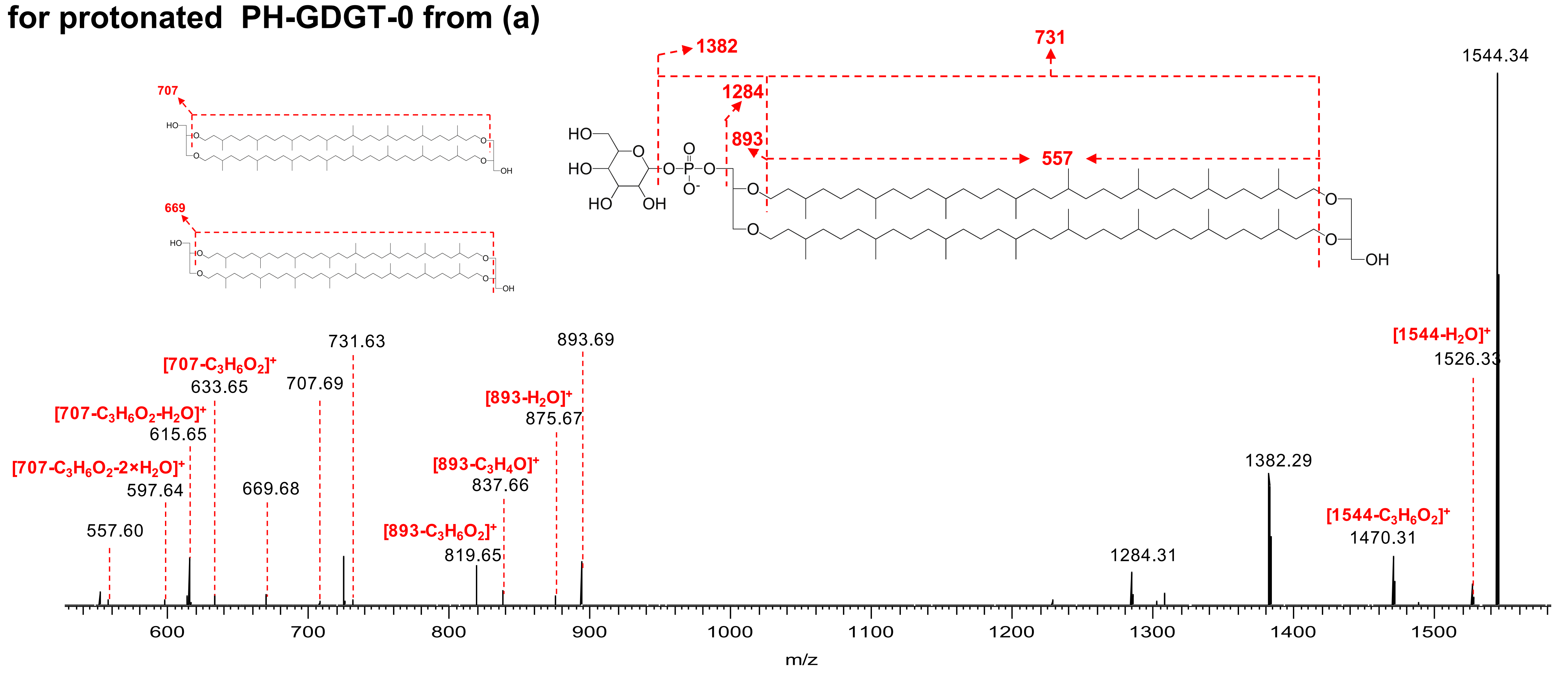

c for protonated PH-GDGTs from sample at 175m of HOT296_S2

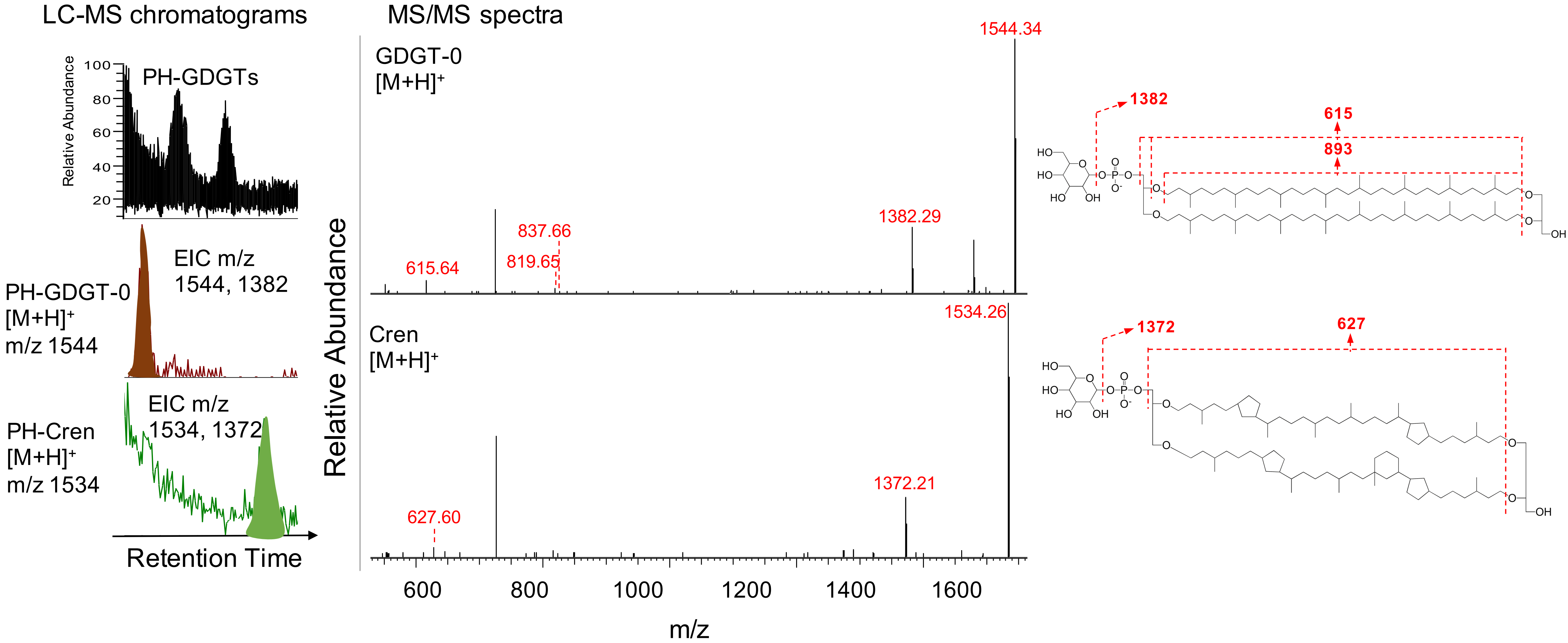

Supplementary Figure 8

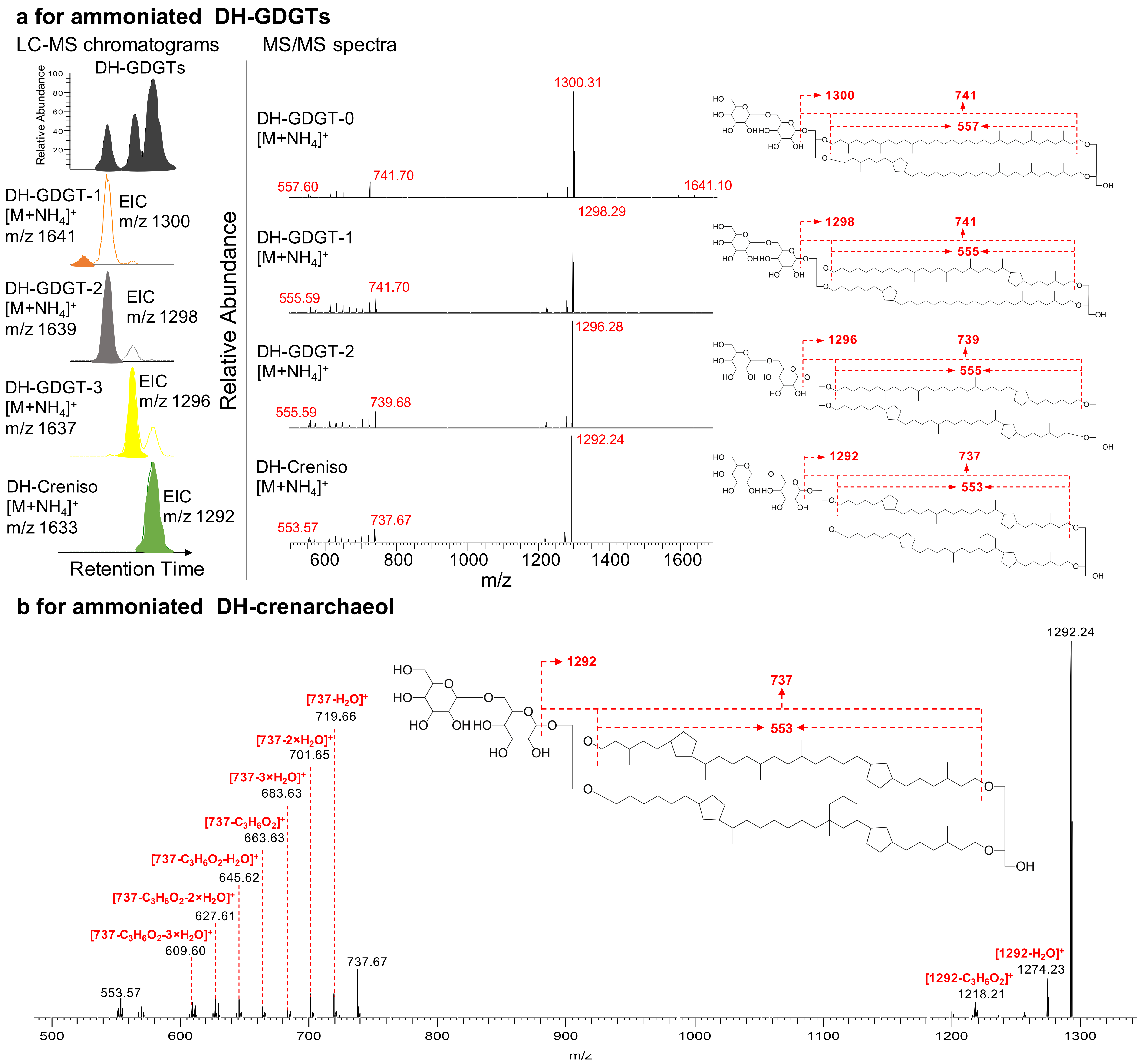

Supplementary Figure 9

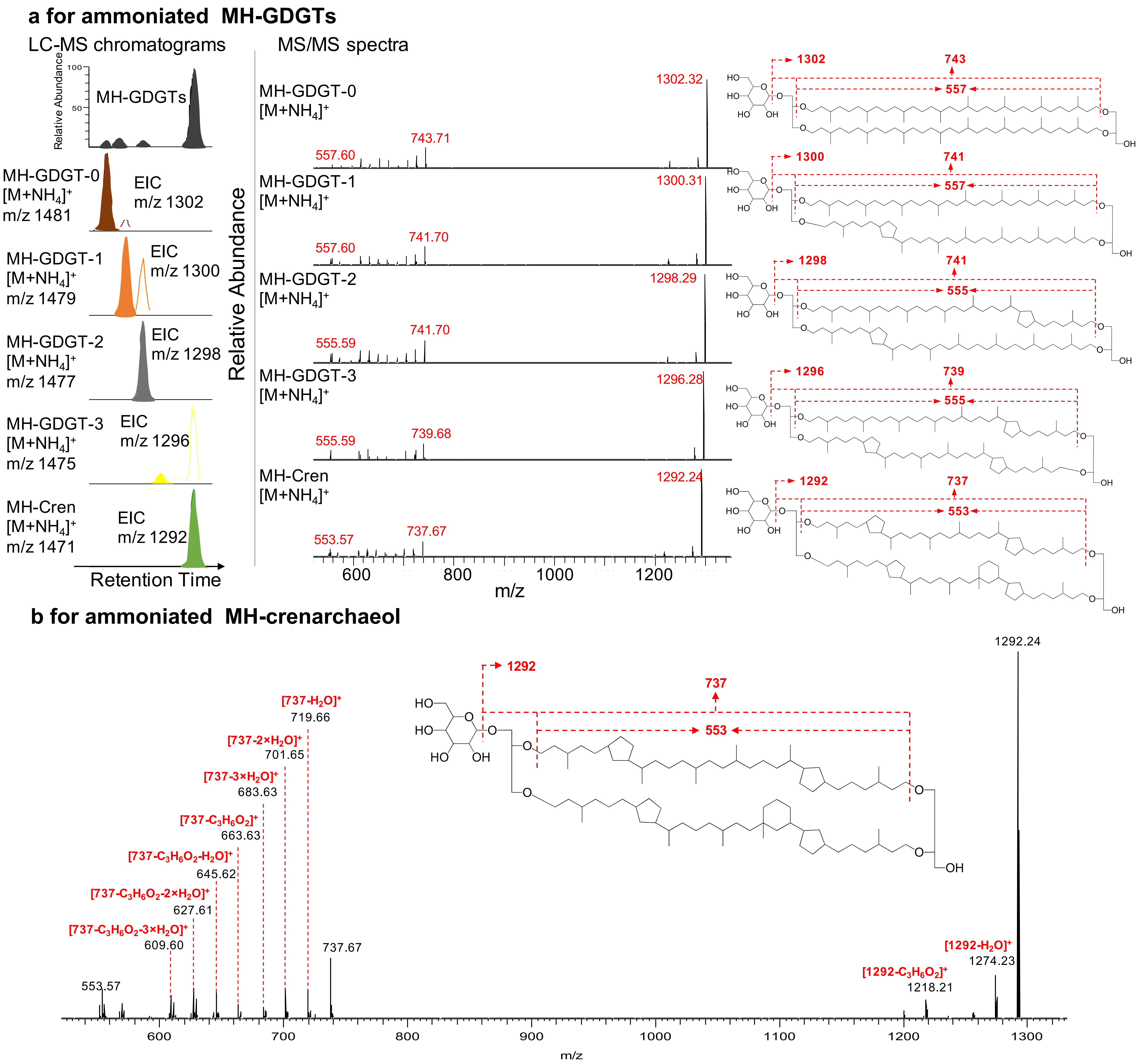

Supplementary Figure 10

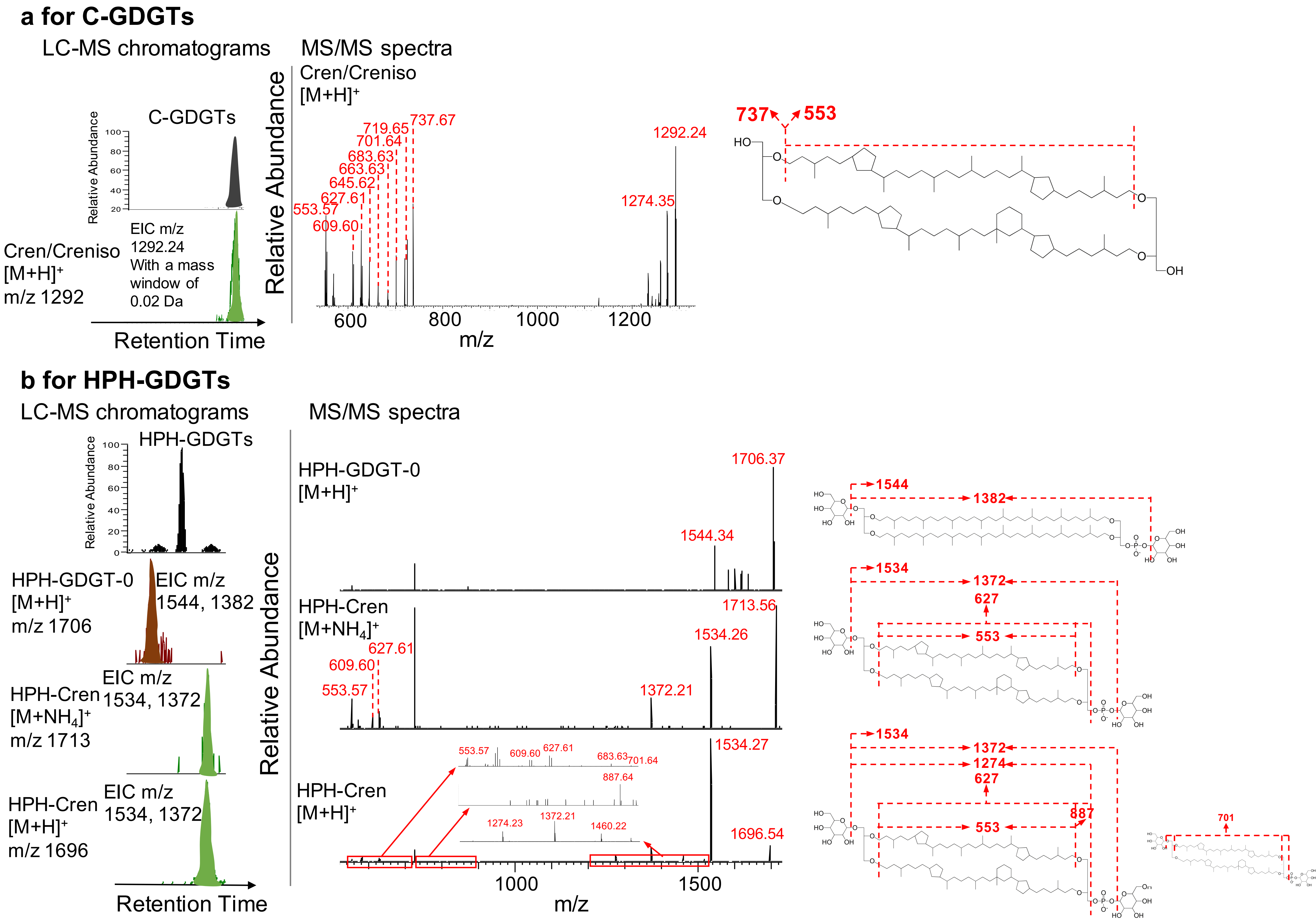

Supplementary Figure 11

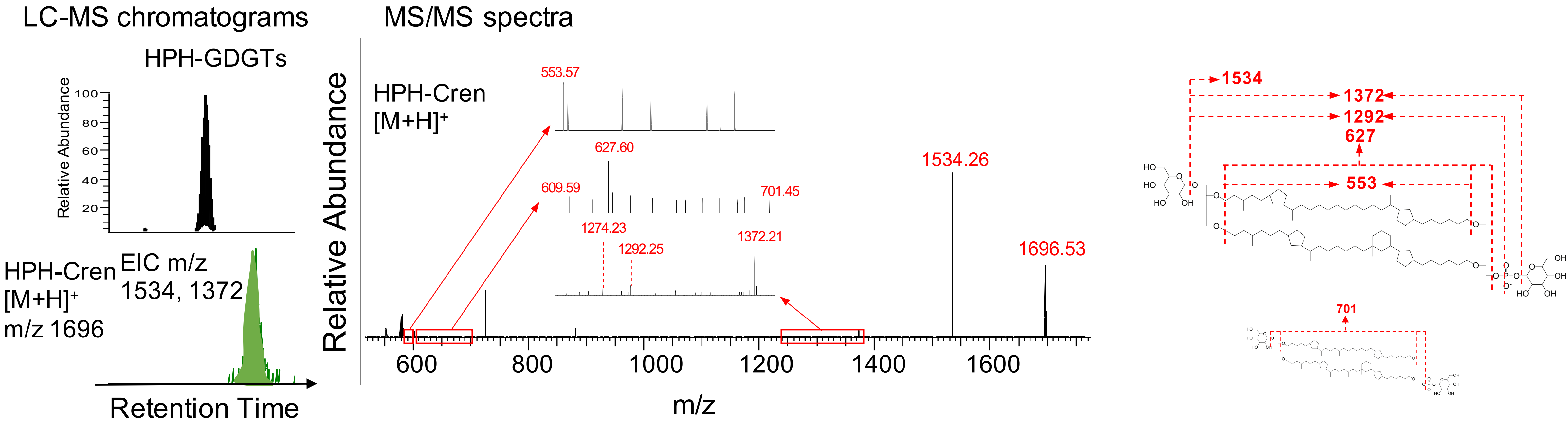

Supplementary Figure 12

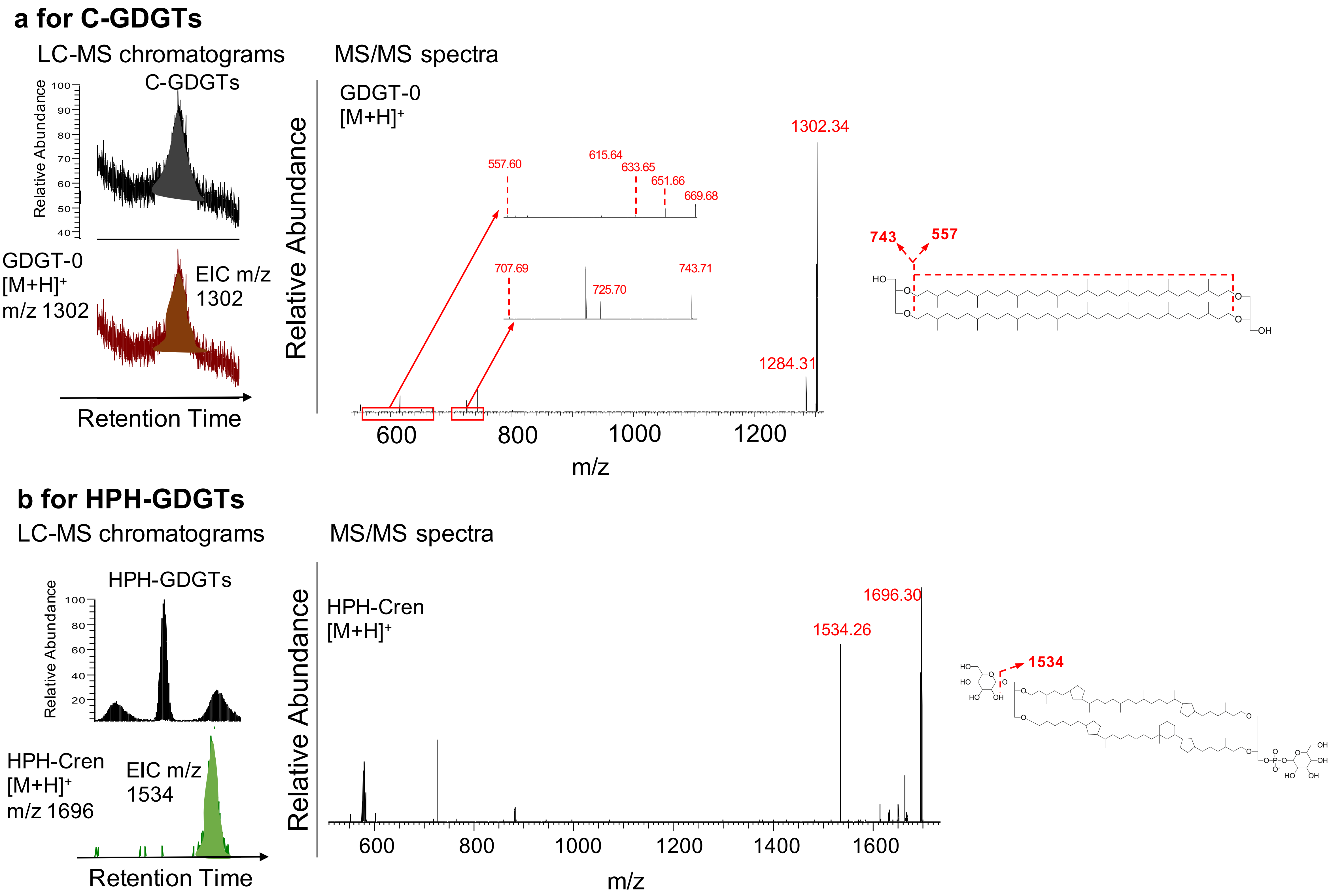

Supplementary Figure 13

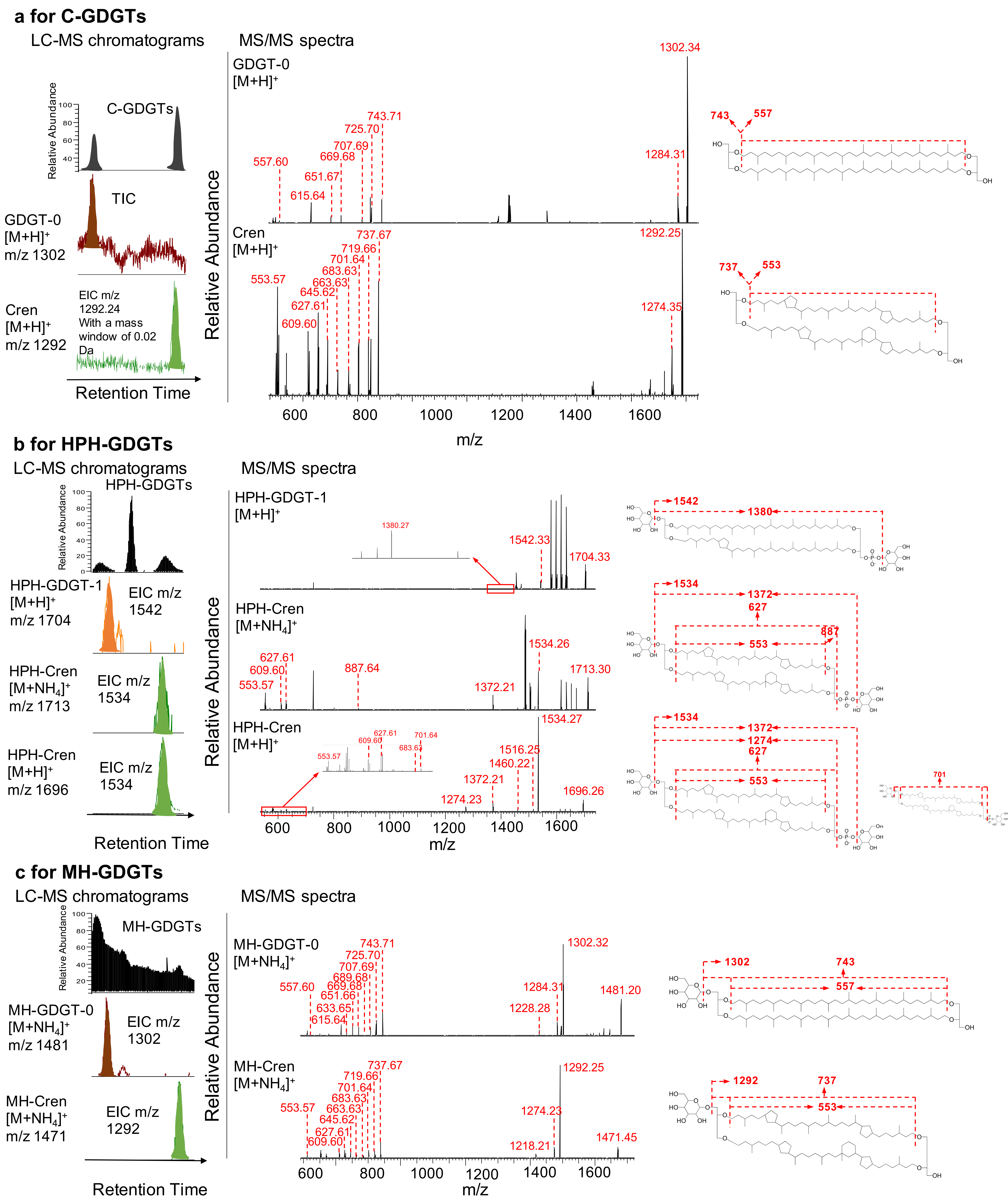

Supplementary Figure 14

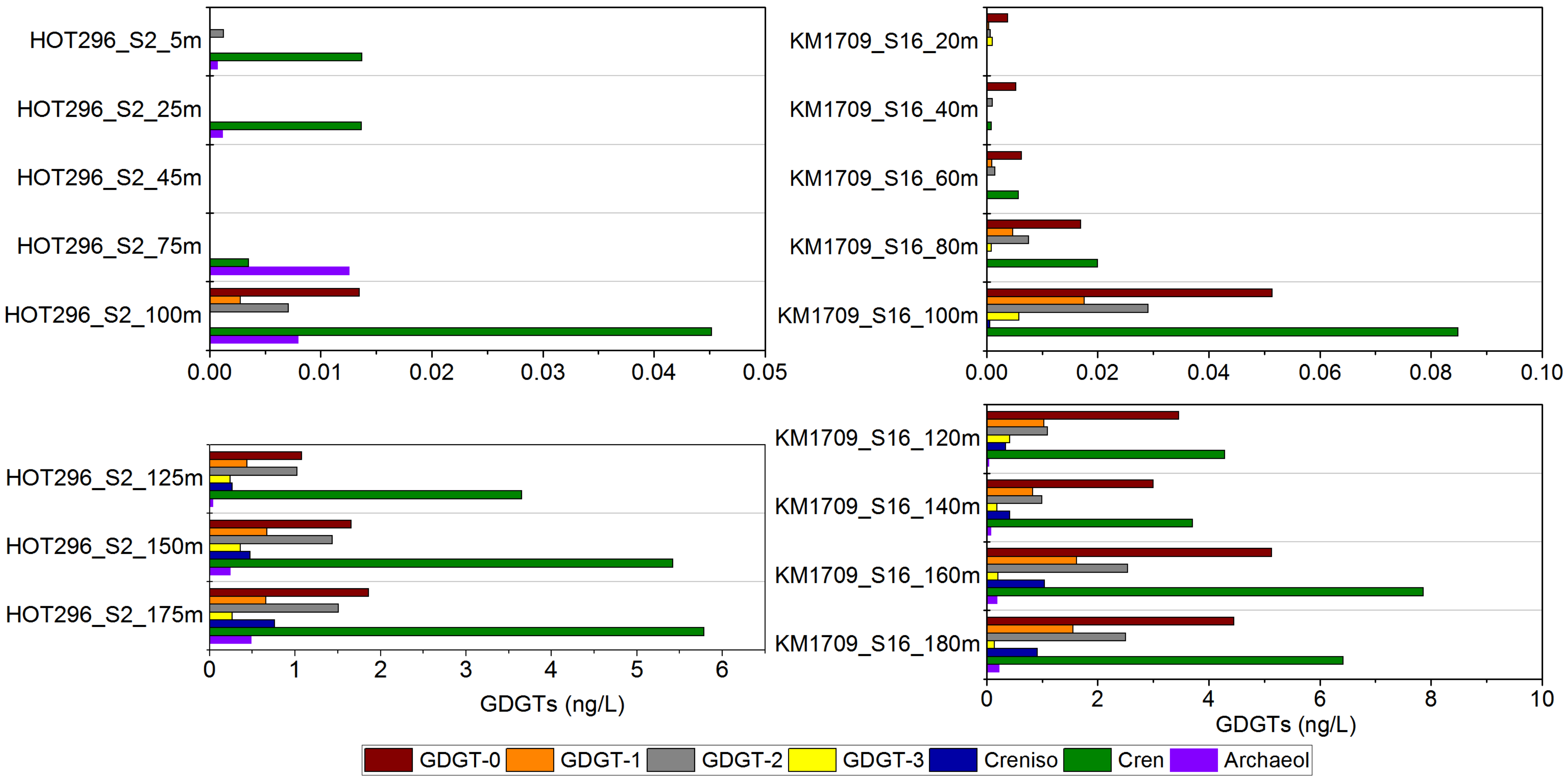

Supplementary Figure 15

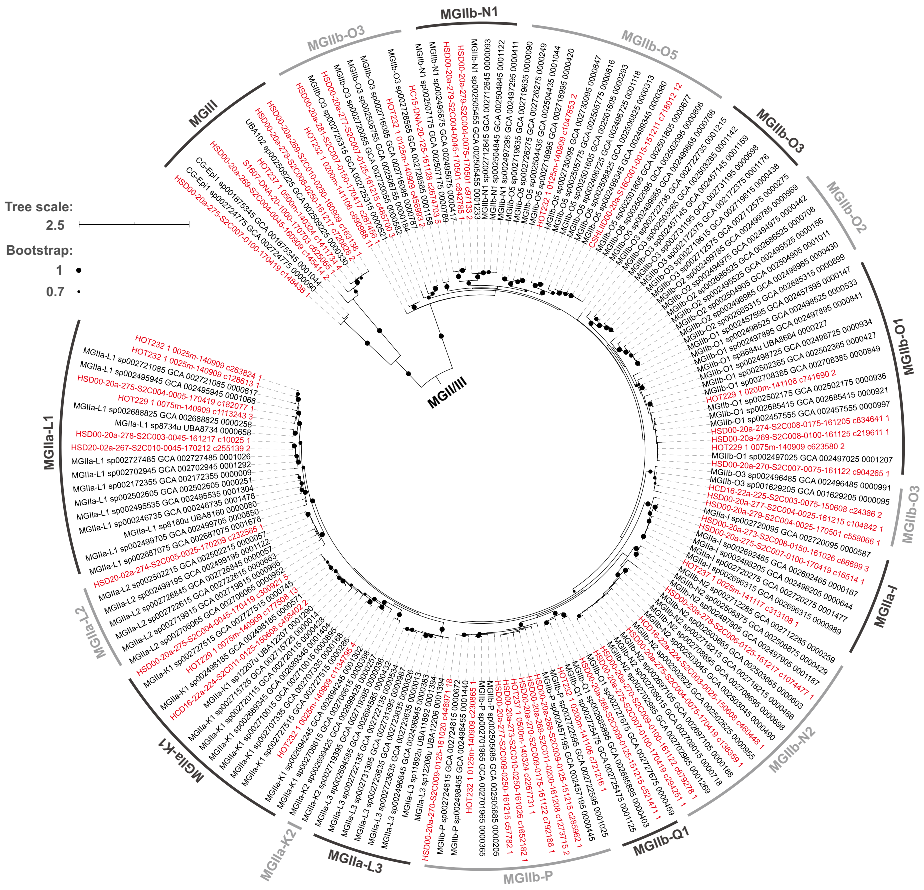

Supplementary Figure 16

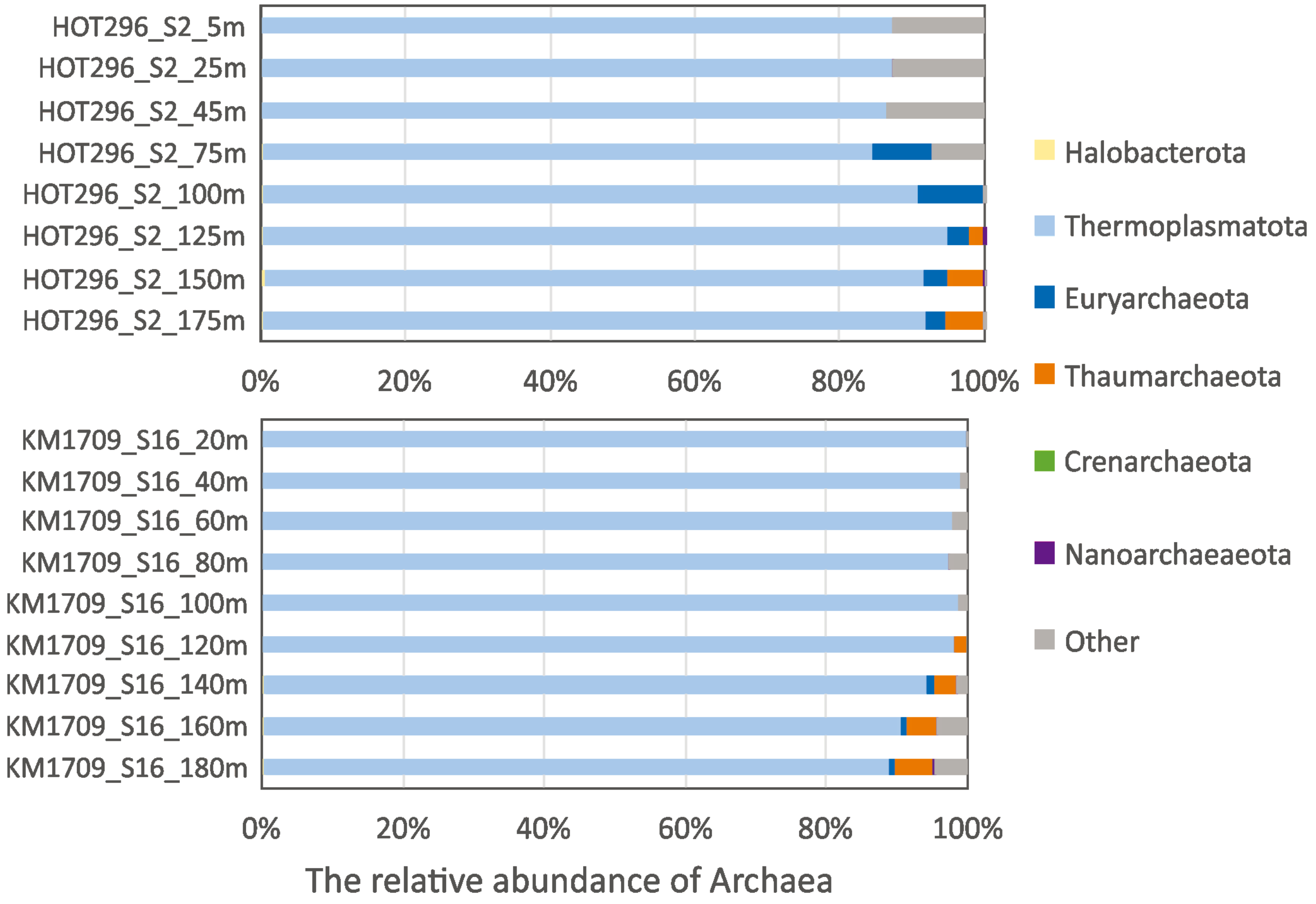

Supplementary Figure 17

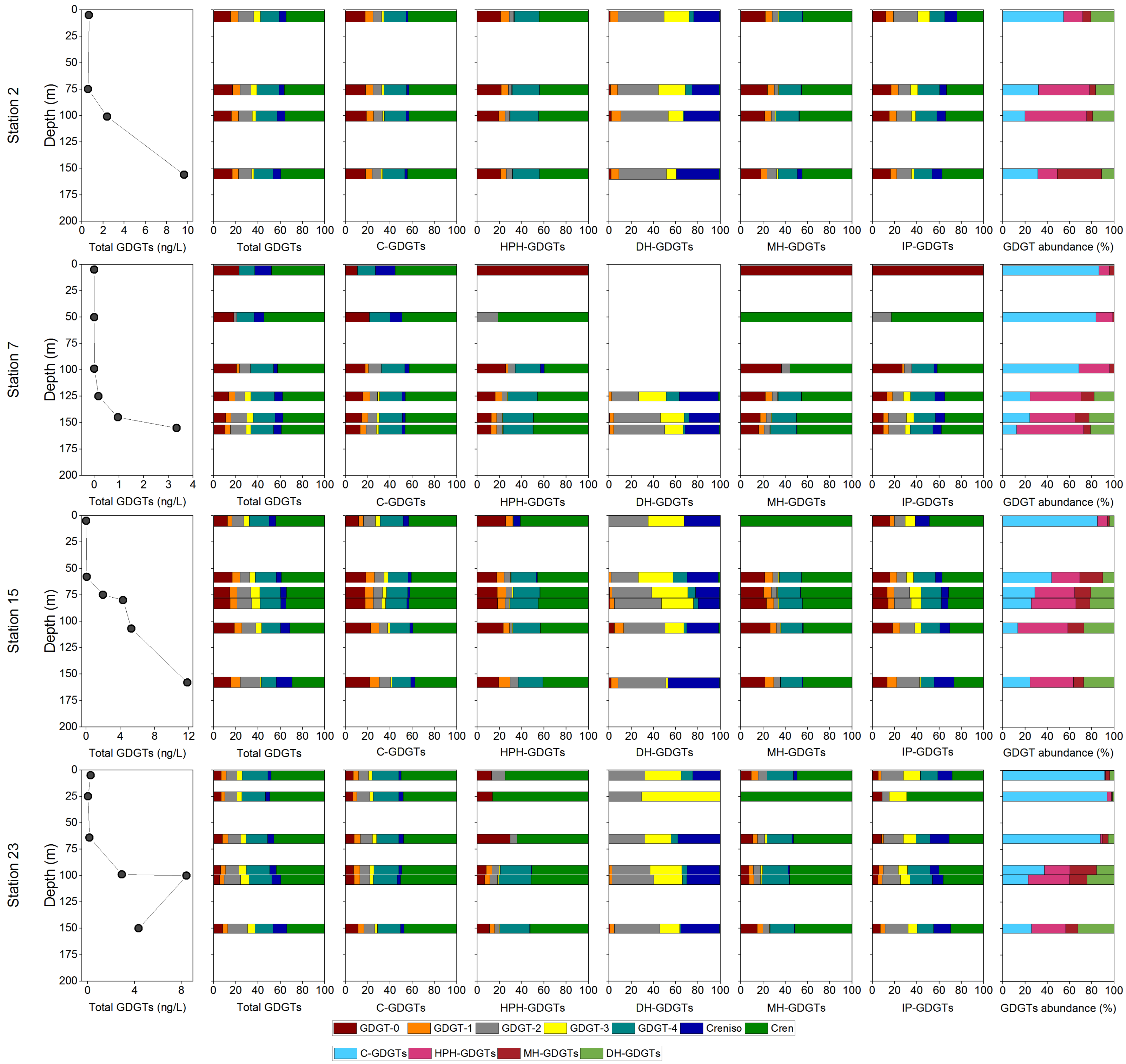

Supplementary Figure 18

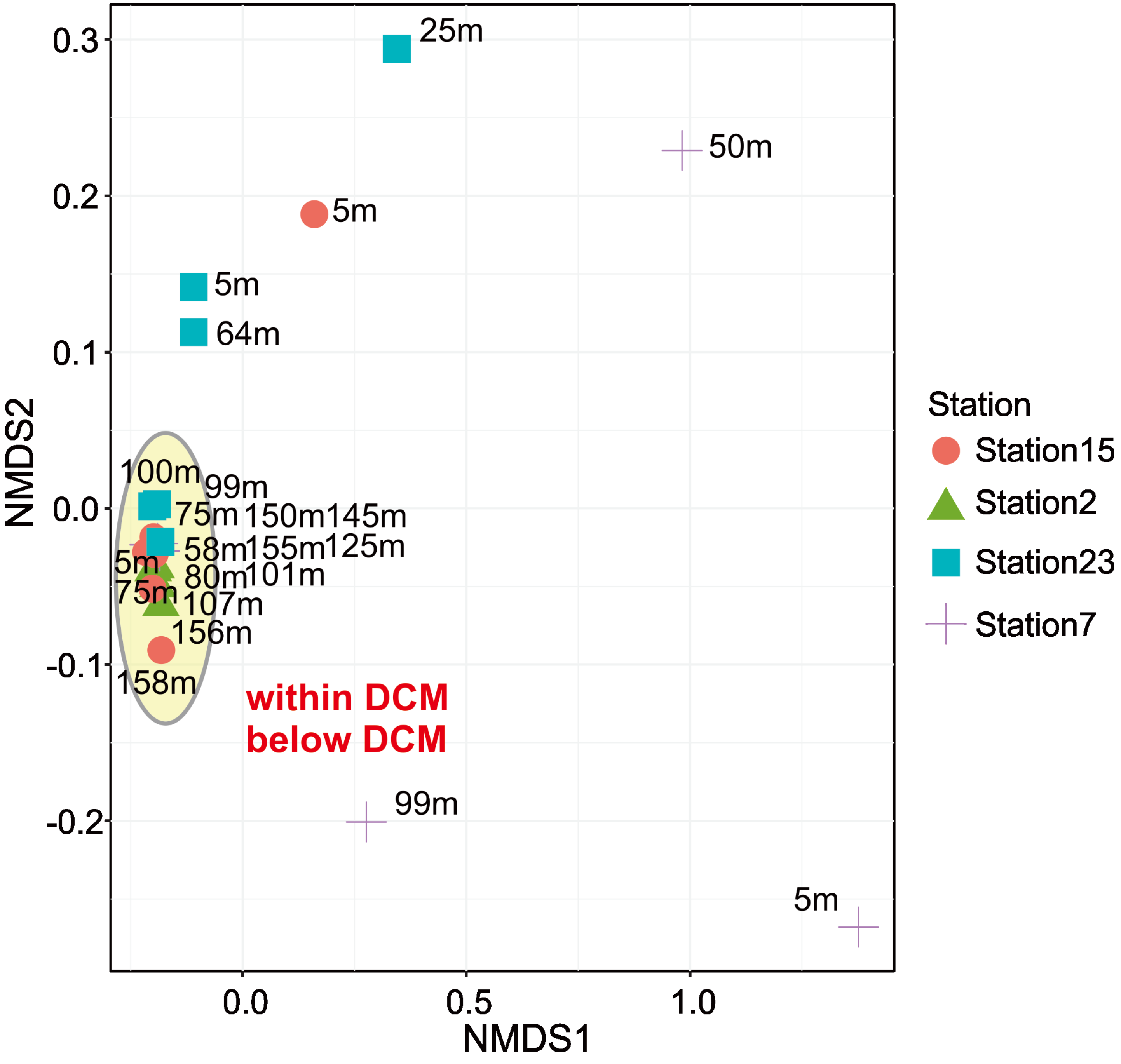

Supplementary Figure 19
