# Supplementary material for: Planktonic Archaeal Ether Lipid Origins in Surface Waters of the North Pacific Subtropical Gyre
Source: Front Microbiol. 2021 Sep 13;12:610675. doi: 10.3389/fmicb.2021.610675 (PMC8473941; doi:10.3389/fmicb.2021.610675)
Supplement: Supplementary Table 1 — Sampling, hydrographic data, archaeal abundance estimates, estimated cellular ether lipid content for Thermoplasmatota, estimated and measured ether lipid concentrations. [file Data_Sheet_2.PDF]

Supplementary Table 1. Sampling, hydographic data, archaeal abundance estimates, estimated cellular ether lipid content for Thermoplasmatota, estimated and measured ether lipid concentrations.

Sample	Date	Cruise	Station	Depth (m)	Temperature (°C)	Salinity (‰)	Density (kg/m3)	Chl a (ug/L)	Cell concentrations (SSU rRNA copies/L)			Measured ether lipid concentrations (ng/L)			Cellular content of ethers for Thermoplasmatota	IP-ethers estimated from Thermoplasmatota	IP-ethers estimated from Thaumarchaeota
									Archaea	Thermoplasmatota	Thaumarchaeota	IP-ethers + C-ethers	C-ethers	IP-ethers[a]			
HOT296_S2_5m	10/7/17	HOT296	S2 (ALOHA)	5	26.75	35.09	22.86	0.07	5.35E+06	5.35E+06	0	0.016	0.017	0.0020	3.66E-10	NA[d]	NA[d]
HOT296_S2_25m	10/7/17	HOT296	S2 (ALOHA)	25	26.75	35.09	22.86	0.07	4.45E+06	4.45E+06	0	0.015	0	0.015	3.34E-09	NA[d]	NA[d]
HOT296_S2_45m	10/7/17	HOT296	S2 (ALOHA)	45	26.76	35.09	22.86	0.08	5.88E+06	5.88E+06	0	0	0	0	NA[b]	NA[d]	NA[d]
HOT296_S2_75m	10/7/17	HOT296	S2 (ALOHA)	75	26.74	35.08	22.87	0.07	6.45E+06	6.43E+06	0	0.016	0.009	0.0074	NA[c]	NA[d]	NA[d]
HOT296_S2_100m	10/7/17	HOT296	S2 (ALOHA)	100	23.57	35.07	23.83	0.15	2.76E+06	2.74E+06	1.15E+04	0.077	0.027	0.049	NA[c]	NA[d]	NA[d]
HOT296_S2_125m	10/7/17	HOT296	S2 (ALOHA)	125	22.62	35.15	24.17	0.17	1.10E+07	7.39E+06	3.56E+06	6.73	0.54	6.18	NA[c]	0.0089	4.52
HOT296_S2_150m	10/7/17	HOT296	S2 (ALOHA)	150	21.7	35.17	24.44	0.08	9.61E+06	5.21E+06	4.29E+06	10.26	0.90	9.36	NA[c]	0.0063	5.45
HOT296_S2_175m	10/7/17	HOT296	S2 (ALOHA)	175	20.92	35.13	24.62	0.02	1.14E+07	6.08E+06	5.22E+06	11.32	1.11	10.21	NA[c]	0.0074	6.63
KM1709_S16_20m	6/2/17	MESO-SCOPE (KM1709)	S16	20	26.13	35.39	23.29	0.12	2.76E+06	2.76E+06	0	0.0058	0.006	0.0020	7.42E-10	NA[d]	NA[d]
KM1709_S16_40m	6/2/17	MESO-SCOPE (KM1709)	S16	40	25.24	35.31	23.51	0.17	4.00E+06	4.00E+06	0	0.0071	0.012	0.0010	2.50E-10	NA[d]	NA[d]
KM1709_S16_60m	6/2/17	MESO-SCOPE (KM1709)	S16	60	22.79	35.18	24.14	0.21	2.11E+06	2.11E+06	0	0.014	0.027	0.0025	1.16E-09	NA[d]	NA[d]
KM1709_S16_80m	6/2/17	MESO-SCOPE (KM1709)	S16	80	19.79	35.02	24.84	0.28	6.02E+06	6.02E+06	0	0.050	0.070	0.0084	1.40E-09	NA[d]	NA[d]
KM1709_S16_100m	6/2/17	MESO-SCOPE (KM1709)	S16	100	18.59	34.95	25.09	0.43	6.70E+06	6.69E+06	1.30E+04	0.19	0.22	0.031	NA[c]	NA[d]	NA[d]
KM1709_S16_120m	6/2/17	MESO-SCOPE (KM1709)	S16	120	17.31	34.81	25.3	0.95	1.19E+07	8.55E+06	3.33E+06	10.67	3.13	7.54	NA[c]	0.010	4.23
KM1709_S16_140m	6/2/17	MESO-SCOPE (KM1709)	S16	140	16.42	34.69	25.42	0.49	7.11E+06	4.28E+06	2.81E+06	9.22	1.48	7.75	NA[c]	0.0052	3.57
KM1709_S16_160m	6/2/17	MESO-SCOPE (KM1709)	S16	160	15.22	34.51	25.56	0.18	1.22E+07	6.34E+06	5.78E+06	18.59	1.67	16.92	NA[c]	0.0077	7.34
KM1709_S16_180m	6/2/17	MESO-SCOPE (KM1709)	S16	180	14.29	34.41	25.68	0.1	1.16E+07	5.10E+06	6.42E+06	16.21	1.90	14.31	NA[c]	0.0062	8.15

Sample	Total ethers (IP-ethers + C-ethers, ng/L)							C-ethers (ng/L)						
	GDGT-0	GDGT-1	GDGT-2	GDGT-3	Creniso	Cren	Archaeol	GDGT-0	GDGT-1	GDGT-2	GDGT-3	Creniso	Cren	Archaeol
HOT296_S2_5m	0	0	0.0012	0	0	0.014	0.00072	0	0	0	0	0	0.017	0
HOT296_S2_25m	0	0	0	0	0	0.014	0.0012	0	0	0	0	0	0	0
HOT296_S2_45m	0	0	0	0	0	0	0	0	0	0	0	0	0	0
HOT296_S2_75m	0	0	0	0	0	0.0035	0.013	0	0	0	0	0	0.0033	0.0053
HOT296_S2_100m	0.013	0.0028	0.0071	0	0	0.045	0.0080	0	0	0	0	0	0.024	0.0032
HOT296_S2_125m	1.08	0.44	1.02	0.24	0.26	3.65	0.035	0.071	0.029	0.050	0.0072	0.017	0.36	0.011
HOT296_S2_150m	1.66	0.67	1.44	0.36	0.47	5.42	0.24	0.12	0.046	0.10	0.022	0.034	0.48	0.093
HOT296_S2_175m	1.86	0.66	1.51	0.26	0.76	5.79	0.49	0.14	0.050	0.12	0.020	0.046	0.53	0.20
KM1709_S16_20m	0.0038	0.00040	0.00064	0.0010	0	0	0	0.0056	0	0	0	0	0	0
KM1709_S16_40m	0.0052	0	0.00100	0	0	0.00085	0	0.0084	0	0	0	0	0.0035	0
KM1709_S16_60m	0.0063	0.00096	0.0015	0	0	0.0057	0	0.011	0	0	0	0	0.016	0
KM1709_S16_80m	0.017	0.0047	0.0075	0.00088	0	0.020	0	0.020	0.0054	0	0	0	0.045	0
KM1709_S16_100m	0.051	0.018	0.029	0.0058	0.00060	0.085	0	0.054	0.015	0.0064	0	0	0.14	0
KM1709_S16_120m	3.46	1.03	1.10	0.41	0.34	4.29	0.04	0.86	0.26	0.28	0.079	0.090	1.53	0.034
KM1709_S16_140m	3.00	0.83	1.00	0.19	0.42	3.71	0.08	0.36	0.11	0.14	0.015	0.048	0.75	0.047
KM1709_S16_160m	5.13	1.62	2.54	0.21	1.04	7.86	0.19	0.38	0.11	0.18	0.017	0.078	0.81	0.086
KM1709_S16_180m	4.45	1.56	2.50	0.14	0.92	6.42	0.23	0.42	0.13	0.23	0.012	0.089	0.93	0.094

[a]The negative value for individual IP-ethers was treated as zero when calculating the total concentration of IP-ethers

[b]Not available for this sample where GDGTs were not detectable. See interpretation in main text.

[c]Not available for calculation of cellular content for Thermoplasmatota ether lipids below 45 m in HOT296_S2 and 80 m in KM1709_S16

[d]Not available for calculation of the IP-ethers estimated from Thermoplasmatota and Thaumarchaeota biomass above DCM

Supplementary Table 2 High quality reads of the archaeal community

	Halobacterota	Thermoplasmatota		Thaumarchaeota			Crenarchaeota	Woesearchaeota
	Halobacteriales	Marine_Group_II	Marine_Group_III	Nitrosopumilales	Marine_Benthic_Group_A_order	Thaumarchaeota_class_order	Aigarchaeales	Woesearchaeota_order
HOT296_S2_5m	0	2668	2168	0	0	0	0	0
HOT296_S2_25m	0	2125	1300	0	0	0	0	3
HOT296_S2_45m	0	2219	1544	0	0	0	0	2
HOT296_S2_75m	23	3867	2657	0	0	0	0	0
HOT296_S2_100m	11	1636	1458	13	0	0	0	0
HOT296_S2_125m	52	3980	3207	3448	0	0	0	14
HOT296_S2_150m	83	3900	3036	5570	146	0	0	64
HOT296_S2_175m	158	12303	9516	17766	1066	16	0	90
KM1709_S16_20m	0	1591	104	0	0	0	0	0
KM1709_S16_40m	0	2022	1453	0	0	0	0	0
KM1709_S16_60m	0	940	872	0	0	0	0	0
KM1709_S16_80m	0	1892	860	0	0	0	0	0
KM1709_S16_100m	0	1529	535	4	0	0	0	0
KM1709_S16_120m	0	4368	1788	2405	7	0	0	0
KM1709_S16_140m	23	3422	2422	3795	43	0	0	0
KM1709_S16_160m	63	8956	5461	12842	267	14	24	102
KM1709_S16_180m	40	4968	3308	10150	229	17	26	97

Supplementary Table 3. The Grs homologs in MGII/III from the Genome Taxonomy Database and ALOHA gene catalogue.

Locus_tag	Target	AAI (%)
MGIIa-I_sp002498205 GCA_002498205_0000644	Saci_1585	31
MGIIa-I_sp002692465 GCA_002692465_0000167	Saci_1585	30
MGIIa-I_sp002696315 GCA_002696315_0000989	Saci_1585	33
MGIIa-I_sp002720095 GCA_002720095_0000587	Saci_1585	31
MGIIa-I_sp002720275 GCA_002720275_0001477	Saci_1585	31
MGIIa-K1_sp002498185 GCA_002498185_0000571	Saci_1585	28
MGIIa-K1_sp002689345 GCA_002689345_0001404	Saci_1585	28
MGIIa-K1_sp002694245 GCA_002694245_0001302	Saci_1585	29
MGIIa-K1_sp002706615 GCA_002706615_0000398	Saci_1585	29
MGIIa-K1_sp002707335 GCA_002707335_0000168	Saci_1585	25
MGIIa-K1_sp002710015 GCA_002710015_0000895	Saci_1585	31
MGIIa-K1_sp002715725 GCA_002715725_0000014	Saci_1585	30
MGIIa-K1_sp002720115 GCA_002720115_0000428	Saci_1585	29
MGIIa-K1_sp002727515 GCA_002727515_0000286	Saci_1585	25
MGIIa-K1_sp002727515 GCA_002727515_0000745	Saci_1585	23
MGIIa-K1_sp12207u UBA12207_0001290	Saci_1585	30
MGIIa-K2_sp002699425 GCA_002699425_0000257	Saci_1585	26
MGIIa-K2_sp002719395 GCA_002719395_0000036	Saci_1585	26
MGIIa-L1_sp000246735 GCA_000246735_0001478	Saci_1585	26
MGIIa-L1_sp002172355 GCA_002172355_0000009	Saci_1585	28
MGIIa-L1_sp002495535 GCA_002495535_0001304	Saci_1585	26
MGIIa-L1_sp002495945 GCA_002495945_0001068	Saci_1585	21
MGIIa-L1_sp002499705 GCA_002499705_0000850	Saci_1585	23
MGIIa-L1_sp002502605 GCA_002502605_0000251	Saci_1585	26
MGIIa-L1_sp002687075 GCA_002687075_0001676	Saci_1585	28
MGIIa-L1_sp002688825 GCA_002688825_0000258	Saci_1585	25
MGIIa-L1_sp002702945 GCA_002702945_0001292	Saci_1585	25
MGIIa-L1_sp002721085 GCA_002721085_0000617	Saci_1585	29
MGIIa-L1_sp002727485 GCA_002727485_0001026	Saci_1585	25
MGIIa-L1_sp8160u UBA8160_0000080	Saci_1585	28
MGIIa-L1_sp8734u UBA8734_0000658	Saci_1585	23
MGIIa-L2_sp002499195 GCA_002499195_0001122	Saci_1585	26
MGIIa-L2_sp002502215 GCA_002502215_0000057	Saci_1585	29
MGIIa-L2_sp002706065 GCA_002706065_0000952	Saci_1585	26
MGIIa-L2_sp002719815 GCA_002719815_0000966	Saci_1585	29
MGIIa-L2_sp002722615 GCA_002722615_0000663	Saci_1585	26
MGIIa-L2_sp002726845 GCA_002726845_0000057	Saci_1585	26
MGIIa-L3_sp002496845 GCA_002496845_0000383	Saci_1585	24
MGIIa-L3_sp002694585 GCA_002694585_0000432	Saci_1585	23
MGIIa-L3_sp002722135 GCA_002722135_0000534	Saci_1585	30
MGIIa-L3_sp002723635 GCA_002723635_0000013	Saci_1585	30
MGIIa-L3_sp002723635 GCA_002723635_0000526	Saci_1585	30
MGIIa-L3_sp002731395 GCA_002731395_0000987	Saci_1585	30
MGIIa-L3_sp11892u UBA11892_0001394	Saci_1585	20
MGIIa-L3_sp12206u UBA12206_0001394	Saci_1585	27
MGIIb-N1_sp002495675 GCA_002495675_0000417	Saci_1585	22

MGIIb-N1_sp002497295	GCA_002497295_0000411	Saci_1585	23
MGIIb-N1_sp002504845	GCA_002504845_0001122	Saci_1585	29
MGIIb-N1_sp002505455	GCA_002505455_0001233	Saci_1585	30
MGIIb-N1_sp002507175	GCA_002507175_0000789	Saci_1585	22
MGIIb-N1_sp002712645	GCA_002712645_0000093	Saci_1585	24
MGIIb-N2_sp002497905	GCA_002497905_0001187	Saci_1585	30
MGIIb-N2_sp002502625	GCA_002502625_0000955	Saci_1585	35
MGIIb-N2_sp002503045	GCA_002503045_0000490	Saci_1585	29
MGIIb-N2_sp002503665	GCA_002503665_0000603	Saci_1585	24
MGIIb-N2_sp002506875	GCA_002506875_0000429	Saci_1585	30
MGIIb-N2_sp002697105	GCA_002697105_0000188	Saci_1585	26
MGIIb-N2_sp002702985	GCA_002702985_0001269	Saci_1585	34
MGIIb-N2_sp002708015	GCA_002708015_0000718	Saci_1585	29
MGIIb-N2_sp002708695	GCA_002708695_0000698	Saci_1585	29
MGIIb-N2_sp002712285	GCA_002712285_0000259	Saci_1585	31
MGIIb-N2_sp002718215	GCA_002718215_0000486	Saci_1585	25
MGIIb-O1_sp002457555	GCA_002457555_0000997	Saci_1585	23
MGIIb-O1_sp002457595	GCA_002457595_0000147	Saci_1585	23
MGIIb-O1_sp002497025	GCA_002497025_0001207	Saci_1585	23
MGIIb-O1_sp002497895	GCA_002497895_0000841	Saci_1585	22
MGIIb-O1_sp002498525	GCA_002498525_0000533	Saci_1585	29
MGIIb-O1_sp002498725	GCA_002498725_0000934	Saci_1585	24
MGIIb-O1_sp002502175	GCA_002502175_0000936	Saci_1585	29
MGIIb-O1_sp002502365	GCA_002502365_0000427	Saci_1585	25
MGIIb-O1_sp002685415	GCA_002685415_0000921	Saci_1585	23
MGIIb-O1_sp002708385	GCA_002708385_0000849	Saci_1585	23
MGIIb-O1_sp8684u	UBA8684_0000227	Saci_1585	23
MGIIb-O2_sp002494975	GCA_002494975_0000442	Saci_1585	28
MGIIb-O2_sp002495525	GCA_002495525_0000156	Saci_1585	28
MGIIb-O2_sp002498985	GCA_002498985_0000430	Saci_1585	21
MGIIb-O2_sp002499785	GCA_002499785_0000969	Saci_1585	30
MGIIb-O2_sp002504905	GCA_002504905_0001011	Saci_1585	34
MGIIb-O2_sp002685315	GCA_002685315_0000899	Saci_1585	29
MGIIb-O2_sp002686525	GCA_002686525_0000708	Saci_1585	28
MGIIb-O3_sp001629205	GCA_001629205_0000095	Saci_1585	26
MGIIb-O3_sp002172375	GCA_002172375_0001176	Saci_1585	22
MGIIb-O3_sp002457145	GCA_002457145_0001159	Saci_1585	21
MGIIb-O3_sp002496485	GCA_002496485_0000991	Saci_1585	31
MGIIb-O3_sp002503285	GCA_002503285_0001142	Saci_1585	21
MGIIb-O3_sp002506755	GCA_002506755_0000184	Saci_1585	23
MGIIb-O3_sp002712575	GCA_002712575_0000275	Saci_1585	21
MGIIb-O3_sp002716085	GCA_002716085_0000787	Saci_1585	31
MGIIb-O3_sp002719615	GCA_002719615_0000438	Saci_1585	23
MGIIb-O3_sp002720055	GCA_002720055_0000582	Saci_1585	25
MGIIb-O3_sp002722735	GCA_002722735_0001215	Saci_1585	23
MGIIb-O3_sp002725315	GCA_002725315_0000521	Saci_1585	21
MGIIb-O3_sp002728565	GCA_002728565_0001158	Saci_1585	22
MGIIb-O3_sp002731195	GCA_002731195_0000698	Saci_1585	20

MGIIb-O5_sp002496725 GCA_002496725_0001118	Saci_1585	20
MGIIb-O5_sp002499345 GCA_002499345_0000380	Saci_1585	22
MGIIb-O5_sp002499865 GCA_002499865_0000768	Saci_1585	22
MGIIb-O5_sp002501605 GCA_002501605_0000293	Saci_1585	27
MGIIb-O5_sp002501805 GCA_002501805_0000677	Saci_1585	22
MGIIb-O5_sp002502095 GCA_002502095_0000806	Saci_1585	20
MGIIb-O5_sp002504435 GCA_002504435_0001044	Saci_1585	24
MGIIb-O5_sp002505775 GCA_002505775_0000816	Saci_1585	28
MGIIb-O5_sp002506825 GCA_002506825_0000313	Saci_1585	22
MGIIb-O5_sp002718995 GCA_002718995_0000420	Saci_1585	24
MGIIb-O5_sp002719635 GCA_002719635_0000090	Saci_1585	28
MGIIb-O5_sp002726275 GCA_002726275_0000249	Saci_1585	27
MGIIb-O5_sp002730095 GCA_002730095_0000847	Saci_1585	28
MGIIb-P_sp002457195 GCA_002457195_0000445	Saci_1585	24
MGIIb-P_sp002498455 GCA_002498455_0001440	Saci_1585	29
MGIIb-P_sp002505685 GCA_002505685_0000205	Saci_1585	29
MGIIb-P_sp002701965 GCA_002701965_0000365	Saci_1585	28
MGIIb-P_sp002722595 GCA_002722595_0001025	Saci_1585	29
MGIIb-P_sp002724815 GCA_002724815_0000672	Saci_1585	29
MGIIb-Q1_sp002685895 GCA_002685895_0000403	Saci_1585	25
MGIIb-Q1_sp002725475 GCA_002725475_0001125	Saci_1585	26
MGIIb-Q1_sp002727675 GCA_002727675_0000459	Saci_1585	29
CG-Epi1_sp001875345 GCA_001875345_0001044	Saci_1585	24
CG-Epi1_sp002724775 GCA_002724775_0000090	Saci_1585	24
UBA102_sp002509225 GCA_002509225_0000330	Saci_1585	24
CSHLIID00-20a-S16C001-0015-151211_c718012_12	Saci_1585	22
HCD16-22a-224-S2C003-0025-150608_c460448_1	Saci_1585	33
HCD16-22a-224-S2C011-0125-150608_c458402_2	Saci_1585	30
HCD16-22a-225-S2C003-0075-150608_c24386_2	Saci_1585	26
HOT229_1_0075m-140909_c177508_13	Saci_1585	28
HOT229_1_0075m-140909_c623580_2	Saci_1585	22
HOT229_1_0075m-140909_c1113243_3	Saci_1585	23
HOT229_1_0200m-141106_c741690_2	Saci_1585	24
HOT232_1_0025m-140909_c128613_1	Saci_1585	28
HOT232_1_0025m-140909_c263824_1	Saci_1585	29
HOT232_1_0025m-140909_c1134795_4	Saci_1585	29
HOT232_1_0125m-140909_c230865_1	Saci_1585	29
HOT232_1_0125m-140909_c456993_2	Saci_1585	30
HOT232_1_0125m-140909_c1047853_2	Saci_1585	24
HOT232_1_0200m-141106_c721216_1	Saci_1585	25
HOT232_1_0200m-141106_c890988_11	Saci_1585	23
HOT237_1_0025m-141117_c313108_1	Saci_1585	31
HOT237_1_0500m-140324_c1411734_4	Saci_1585	26
HOT237_1_0500m-140324_c2267731_1	Saci_1585	29
HSD00-20a-268-S2C009-0125-151215_c285962_1	Saci_1585	25
HSD00-20a-268-S2C009-0125-151215_c521471_1	Saci_1585	22
HSD00-20a-268-S2C011-0200-161208_c1273715_2	Saci_1585	23
HSD00-20a-269-S2C004-0045-160909_c145414_2	Saci_1585	25

HSD00-20a-269-S2C008-0100-161125_c219611_1	Saci_1585	27
HSD00-20a-269-S2C010-0250-160909_c163138_1	Saci_1585	24
HSD00-20a-270-S2C007-0075-161122_c904265_1	Saci_1585	29
HSD00-20a-270-S2C009-0100-161122_c679278_1	Saci_1585	34
HSD00-20a-270-S2C009-0125-161020_c448971_18	Saci_1585	29
HSD00-20a-270-S2C009-0175-161122_c792186_1	Saci_1585	32
HSD00-20a-273-S2C008-0150-161026_c86699_3	Saci_1585	21
HSD00-20a-273-S2C010-0250-161026_c1652182_1	Saci_1585	30
HSD00-20a-274-S2C008-0175-161205_c834641_1	Saci_1585	23
HSD00-20a-275-S2C004-0005-170419_c182077_1	Saci_1585	24
HSD00-20a-275-S2C004-0045-170419_c300921_5	Saci_1585	30
HSD00-20a-275-S2C004-0075-170419_c138359_1	Saci_1585	34
HSD00-20a-275-S2C007-0100-170419_c16514_1	Saci_1585	31
HSD00-20a-275-S2C007-0100-170419_c148438_1	Saci_1585	24
HSD00-20a-275-S2C007-0100-170419_c294251_1	Saci_1585	31
HSD00-20a-277-S2C004-0025-161215_c104842_1	Saci_1585	24
HSD00-20a-277-S2C007-0125-161215_c485700_3	Saci_1585	20
HSD00-20a-277-S2C009-0250-161215_c57782_1	Saci_1585	29
HSD00-20a-278-S2C003-0045-161217_c10025_1	Saci_1585	27
HSD00-20a-278-S2C006-0125-161217_c1074477_1	Saci_1585	28
HSD00-20a-278-S2C008-0250-161217_c720828_2	Saci_1585	23
HSD00-20a-279-S2C004-0025-170501_c558066_1	Saci_1585	24
HSD00-20a-279-S2C004-0045-170501_c842785_1	Saci_1585	22
HSD00-20a-279-S2C004-0075-170501_c97133_2	Saci_1585	27
HSD00-20a-281-S2C007-0150-170417_c287456_1	Saci_1585	23
HSD20-02a-267-S2C010-0045-170212_c255139_2	Saci_1585	25
HSD20-02a-274-S2C005-0025-170209_c232565_1	Saci_1585	28
HC15-DNA-20-125-161128_c2674703_5	Saci_1585	21
S1607-DNA-20-1000-170703_c625065_1	Saci_1585	23

Supplementary Table 4. The count profile of Rps3 sequences from the ALOHA gene catalogue at the surface of Station ALOHA.

			Counts in metagenomic data[a]		
	Depth (m)	Date	Marine_Group_III	Marine_Group_II	Nitrososphaerales
HSD00-20a-267-S2C010-0005-151221	5	11/2014	0	5	0
HSD00-20a-267-S2C010-0005-161208	5	11/2014	1	13	0
HSD00-20a-268-S2C004-0005-151215	5	12/2014	0	2	0
HSD00-20a-269-S2C004-0005-160909	5	2/2015	3	4	0
HSD00-20a-269-S2C004-0005-161125	5	2/2015	2	3	0
HSD00-20a-270-S2C007-0005-161020	5	3/2015	0	5	0
HSD00-20a-270-S2C007-0005-161122	5	3/2015	2	5	0
HSD00-20a-271-S2C004-0005-161007	5	4/2015	0	4	0
HSD00-20a-272-S2C005-0005-161221	5	5/2015	0	3	0
HSD00-20a-273-S2C005-0005-161026	5	6/2015	0	2	0
HSD00-20a-275-S2C004-0005-170419	5	8/2015	0	8	0
HSD00-20a-277-S2C004-0005-161215	5	10/2015	0	2	0
HSD00-20a-278-S2C003-0005-161217	5	11/2015	1	4	0
HSD00-20a-279-S2C004-0005-170501	5	12/2015	4	10	0
HSD00-20a-280-S2C004-0005-170508	5	1/2016	0	6	0
HSD00-20a-281-S2C004-0005-170417	5	2/2016	0	5	0
HSD00-20a-282-S2C004-0005-170504	5	3/2016	1	5	0
HSD00-20a-283-S2C004-0005-170428	5	4/2016	0	1	0
HSD00-20a-267-S2C010-0025-151215	25	11/2014	1	8	0
HSD00-20a-268-S2C004-0025-151124	25	12/2014	0	2	0
HSD00-20a-268-S2C004-0025-151215	25	12/2014	0	4	0
HSD00-20a-269-S2C004-0025-160909	25	2/2015	0	6	0
HSD00-20a-269-S2C004-0025-161125	25	2/2015	0	4	0
HSD00-20a-270-S2C007-0025-161020	25	3/2015	7	17	0
HSD00-20a-270-S2C007-0025-161122	25	3/2015	3	16	0
HSD00-20a-271-S2C004-0025-161007	25	4/2015	0	1	0
HSD00-20a-272-S2C005-0025-161221	25	5/2015	0	1	0
HSD00-20a-273-S2C005-0025-161026	25	6/2015	0	6	0
HSD00-20a-274-S2C005-0025-161205	25	7/2015	0	6	0
HSD00-20a-275-S2C004-0025-170419	25	8/2015	0	1	0
HSD00-20a-277-S2C004-0025-161215	25	10/2015	1	3	0
HSD00-20a-278-S2C003-0025-161217	25	11/2015	1	9	0
HSD00-20a-279-S2C004-0025-170501	25	12/2015	4	13	0
HSD00-20a-280-S2C004-0025-170508	25	1/2016	0	2	0
HSD00-20a-281-S2C004-0025-170417	25	2/2016	1	4	0
HSD00-20a-282-S2C004-0025-170504	25	3/2016	1	2	0
HSD00-20a-283-S2C004-0025-170428	25	4/2016	2	12	0

[a]The abundance of key archaeal lineages was calculated using phylogenetic tree insertion of open reading frames derived from individual metagenomic reads into a preconstructed ribosomal protein S3 (RPS3) marker tree using graftM v0.13.1(Boyd et al., 2018). The preconstructed phylogenetic tree was constructed using all RPS3 protein sequences derived from GTDB v89(Parks et al., 2018) clustered at 99% amino acid identity using cd-hit v4.8.1 (Fu et al., 2012).

References:

Boyd, J.A., Woodcroft, B.J., and Tyson, G.W. (2018) GraftM: a tool for scalable, phylogenetically informed classification of genes within metagenomes. Nucleic Acids Research 46, e59-e59.

Fu, L., Niu, B., Zhu, Z., Wu, S. and Li, W. (2012) CD-HIT: accelerated for clustering the next generation sequencing data. Bioinformatics 28: 3150-3152.

Parks, D.H., Chuvochina, M., Waite, D.W., Rinke, C., Skarshewski, A., Chaumeil, P.A. and Hugenholtz, P. (2018) A standardized bacterial taxonomy based on genome phylogeny substantially revises the tree of life. Nature biotechnology 36: 996-1004.
