# Supplementary material for: Planktonic Archaeal Ether Lipid Origins in Surface Waters of the North Pacific Subtropical Gyre
Source: Front Microbiol. 2021 Sep 13;12:610675. doi: 10.3389/fmicb.2021.610675 (PMC8473941; doi:10.3389/fmicb.2021.610675)
Supplement: Supplementary file 3 [file Data_Sheet_1.docx]

**Supplementary Text**

**Identification of core and intact polar lipids**

HPH-, PH-, DH-, MH- and C-GDGTs and C-archaeol (Suppl. Fig. 1) were identified by UHPLC-ESI-MS_Q Exactive_ under reversed-phase conditions, and their characteristic fragment ions in MS/MS spectra were detected on samples at 120 and 140 m in KM1709_S16 and 175 m in HOT296_S2 (Suppl. Fig. 5 to 9). Core lipids were analyzed in both non-hydrolyzed and acid-hydrolyzed fractions. C-GDGTs with less polarity were the last eluting lipids with ammoniated [M+NH_4_]^+^, sodiated [M+Na]^+^ and protonated [M+H]^+^ molecules detected in MS spectra. Their MS/MS spectra were characterized by the protonated molecule [M+H]^+^ and product ions that resulted from loss of H_2_O, glycerol and/or biphytane moieties (Suppl. Fig. 5). C-archaeol was the first eluting compound with all three ions determined as well, the MS/MS fragmentation of which was characterized by product ion caused by loss of one phytane unit and the molecule [M+NH_4_]^+^ or [M+H]^+^ (Suppl. Fig. 6).

The second eluting compounds were HPH-GDGTs with ions [M+NH_4_]^+^, [M+Na]^+^ and [M+H]^+^ determined in MS spectra. The characteristic fragments in MS/MS spectra included product ions caused by loss of H_2_O, glycerol, hexose, the whole hexose-phosphate-hexose head group and/or biphytane units (Suppl. Fig. 7). PH-GDGTs were the third eluting compounds with only ion [M+H]^+^ containing SN ≥ 5 in MS spectra (Suppl. Fig. 8). The MS/MS fragmentation was conducted in PH-GDGTs enriched sample that was collected from sediment trap at 4000 m in station ALOHA. All precursors [M+NH_4_]^+^, [M+Na]^+^ and [M+H]^+^ were detected in the sediment trap sample and MS/MS fragmentation ions were characterized by the molecule [M+H]^+^ and product ions obtained from loss of H_2_O, glycerol, hexose, phosphate-hexose head group and/or biphytane units (Suppl. Fig. 8). We performed MS/MS fragmentation on the sample at 175 m of HOT296_S2, which yielded fewer fragment ions (Suppl. Fig. 8). After the lipid cluster of PH-GDGTs, DH- and MH-GDGTs were eluted sequentially with ions [M+NH_4_]^+^ and [M+Na]^+^ detected in SN ≥ 5. The fragments in MS/MS spectra consisted of ions produced from loss of one (or two) hexose unit(s), H_2_O and/or glycerol (Suppl. Fig. 9 and 10).
